# Gene-Expression Profiling Suggests Impaired Signaling via the Interferon Pathway in *Cstb^-/-^* Microglia

**DOI:** 10.1371/journal.pone.0158195

**Published:** 2016-06-29

**Authors:** Inken Körber, Shintaro Katayama, Elisabet Einarsdottir, Kaarel Krjutškov, Paula Hakala, Juha Kere, Anna-Elina Lehesjoki, Tarja Joensuu

**Affiliations:** 1 Folkhälsan Institute of Genetics, Helsinki, Finland; 2 Research Program’s Unit, Molecular Neurology, University of Helsinki, Helsinki, Finland; 3 Neuroscience Center, University of Helsinki, Helsinki, Finland; 4 Department of Biosciences and Nutrition, Karolinska Institutet, Stockholm, Sweden; 5 Competence Centre on Health Technologies, Tartu, Estonia; Icahn School of Medicine at Mount Sinai, UNITED STATES

## Abstract

Progressive myoclonus epilepsy of Unverricht-Lundborg type (EPM1, OMIM254800) is an autosomal recessive neurodegenerative disorder characterized by stimulus-sensitive and action-activated myoclonus, tonic-clonic epileptic seizures, and ataxia. Loss-of-function mutations in the gene encoding the cysteine protease inhibitor cystatin B (CSTB) underlie EPM1. The deficiency of CSTB in mice (*Cstb*^*-/-*^ mice) generates a phenotype resembling the symptoms of EPM1 patients and is accompanied by microglial activation at two weeks of age and an upregulation of immune system-associated genes in the cerebellum at one month of age. To shed light on molecular pathways and processes linked to CSTB deficiency in microglia we characterized the transcriptome of cultured *Cstb*^*-/-*^ mouse microglia using microarray hybridization and RNA sequencing (RNA-seq). The gene expression profiles obtained with these two techniques were in good accordance and not polarized to either pro- or anti-inflammatory status. In *Cstb*^*-/-*^ microglia, altogether 184 genes were differentially expressed. Of these, 33 genes were identified by both methods. Several interferon-regulated genes were weaker expressed in *Cstb*^*-/-*^ microglia compared to control. This was confirmed by quantitative real-time PCR of the transcripts *Irf7* and *Stat1*. Subsequently, we explored the biological context of CSTB deficiency in microglia more deeply by functional enrichment and canonical pathway analysis. This uncovered a potential role for CSTB in chemotaxis, antigen-presentation, and in immune- and defense response-associated processes by altering JAK-STAT pathway signaling. These data support and expand the previously suggested involvement of inflammatory processes to the disease pathogenesis of EPM1 and connect CSTB deficiency in microglia to altered expression of interferon-regulated genes.

## Introduction

The neurodegenerative disease progressive myoclonus epilepsy of Unverricht-Lundborg type (EPM1, OMIM254800) is an autosomal recessive disease with onset between 6 and 16 years of age. It is characterized by progressive, stimulus-sensitive, and action-activated myoclonus, which is resistant to medication and severely impairs patients’ everyday life [1]. In addition, the patients have tonic-clonic epileptic seizures and develop ataxia. Loss-of-function mutations in the cystatin B (*CSTB*) gene that encodes the cysteine protease inhibitor CSTB [2] underlie EPM1. The mutations are associated with reduced *CSTB* mRNA as well as protein expression [3, 4]. CSTB is a cytoplasmic protein, enriched around lysosomes, and in undifferentiated cells, it has been detected also in nuclei [5]. The increased proteolytic activity of cathepsin B identified in CSTB-deficient cerebellar granule neurons and of cathepsin B, L, and S in EPM1 patient lymphoblastoid cells implies that CSTB functions as a cathepsin inhibitior *in vivo* [6, 7]. CSTB has been linked to the protection of neurons from apoptosis [8] and oxidative stress [6], as well as regulation of cell cycle entry [9]. However, the underlying molecular mechanisms and how the loss of CSTB causes the phenotype of EPM1 remain unknown.

The CSTB-deficient (*Cstb*^*-/-*^) mouse [8] is a widely used model to study EPM1 disease pathogenesis [10–14]. Its phenotype includes the characteristic symptoms of EPM1 patients: the mice develop myoclonus by one month and progressive ataxia by six months of age [8]. Consistent with findings in human patients [15–17], there is progressive atrophy, cortical thinning, and neuron and white matter loss in the brain of *Cstb*^*-/-*^ mice affecting particularly the cerebellum and the thalamocortical system [8, 14, 16]. The earliest neuropathological finding in *Cstb*^*-/-*^ mice is the activation of microglia, the resident tissue macrophages of the CNS, at two weeks of age. This is followed by the activation of astrocytes, myoclonus, and progressive neuronal degeneration from one month onwards [14]. Gene-expression profiling has revealed an upregulation of genes associated with immune-system processes in the cerebellum of *Cstb*^*-/-*^ mice at one month, and led to the discovery of alterations in GABAergic signaling already at one week of age [18]. In detail, findings implying a diminished number of GABAergic pre- and postsynaptic terminals, decreased inhibition, and reduced ligand binding to the α_6_-subunit-containing GABA_A_ receptors were identified in the cerebellum of *Cstb*^*-/-*^ mice [18].

Interestingly, the morphology of activated microglia in young *Cstb*^*-/-*^ mice resembles that of phagocytic brain macrophages and they develop thickened, branched processes at six months of age [14]. Moreover, the activation of microglia in *Cstb*^*-/-*^ mouse brain is dysfunctional, which manifests as an imbalance between pro-inflammatory and anti-inflammatory microglial polarization and as increased chemokine release and chemotactic activity, but possibly reduced antigen presentation and phagocytic capacity [19]. In order to gain insight into the molecular mechanisms underlying the abnormal microglial activation, we studied the transcriptional profile of *Cstb*^*-/-*^ mouse microglia. Our data link CSTB deficiency to downregulation of the interferon-signaling pathway in microglia.

## Materials and Methods

### Ethics statement

The animal research protocols were approved by the Animal Ethics Committee of the State Provincial Office of Southern Finland (decision no. STU376A, STH660A, and STH524A).

### Mice

*Cstb*^*-/-*^ mice were obtained from The Jackson Laboratory (Bar Harbor, ME; 129-*Cstb*^*tm1Rm*^/J; stock no. 003486) [8]. Age-matched wild-type mice of the same background were used as controls. The mice were housed in the Center for laboratory animals at the University of Helsinki with a 12 h / 12 h light-dark cycle and access to food and water ad libitum.

### Primary microglial cultures

Cultures of mixed glia cells were extracted from postnatal day 5 (P5) mice as described earlier [20] with slight modifications. Briefly, the P5 mice were euthanized carefully and without delay by decapitation and after the removal of meninges, the cortices were triturated and incubated with 20% trypsin (TrypLE Express, Life technologies, Carlsbad, CA) and 20 μg DNase (Roche Diagnostics, Basel, Switzerland) for 20 min at 37°C, 5% CO_2_. Next, the cells were triturated 20 times, centrifuged (5 min at 1000 rpm), and, after resuspension, plated in fresh growth medium (DMEM / 2 mM L-glutamine / 10% FCS / 1% penicillin-streptomycin) to 1% poly-D-lysine (PDL) (Sigma-Aldrich, St. Louis, MO) coated T-75 tissue culture flasks. After shaking off the primary microglia from the mixed glia cells at confluency (225 rpm, 37°C for 2.5 h), the cells were re-plated and the growth medium was changed after 1 h at 37°C, 5% CO_2_ to fresh medium containing 5 ng/ml mouse macrophage-colony stimulating factor (M-CSF, R&D Systems, Minneapolis, MN). The purity of microglial cultures was assessed by indirect immunofluorescence with antibodies raised against the microglial marker F4/80 (AbD Serotec, Hercules, CA) and the astrocytic marker glial fibrillary acidic protein (GFAP) (DakoCytomation, Santa Clara, CA).

### RNA sample preparation for gene-expression profiling

Total RNA was extracted from cultured microglia of ten control and ten *Cstb*^*-/-*^ mice using the PerfectPure RNA Cultured Cell kit (5 PRIME, Hilden, Germany). For each sample (five control and five *Cstb*^*-/-*^ total RNA samples) the microglia cells of two mice of the same genotype were pooled and the extracted RNA quality was controlled by chip-based capillary electrophoresis (Bioanalyzer 2100, Agilent, Santa Clara, CA). All samples had an RNA integrity number (RIN) > 7.5.

### Transcriptome analysis by microarrays

Synthesis of cRNA from 100 ng total RNA per each of the five *Cstb*^*-/-*^ and control microglial samples and its hybridization to GeneChip Mouse Exon 1.0 ST arrays (Affymetrix Inc., Santa Clara, CA, USA) were performed at the Helsinki Biomedicum Biochip Center (Finland). The quality of each microarray was assessed by the Affymetrix GeneChip Operating Software (GCOS) (Affymetrix Inc.) and the raw data were imported into GeneSpring GX software Version 12 (Silicon Genetics, Incorporated, Redwood City, CA, USA) as an exon expression experiment. The raw data were pre-processed by the RMA16 algorithm [21] considering only probes with the highest confidence level (‘core’), which are based on Reference Sequence (RefSeq) and full-length Genbank mRNAs as annotation sources. Probe summarization of the data was performed by the RMA algorithm, which includes background correction, normalization, and gene-level summarization. Shortly, the background was adjusted on a per-chip basis and the normalization on probe level was performed by quantile normalization. The probe level data of the perfect match (PM) values are summarized to exon-level probe sets and further to gene-level transcript clusters, resulting in one expression value for each gene covered by the array. Replicate samples of the same genotype were analysed in groups. Quality control on sample level was performed by principal component analysis (PCA) and two arrays (one control and one *Cstb*^*-/-*^ array), which were outliers, were removed from downstream analysis. The expression values of the genes were filtered based on the summarized expression data; the lowest 20^th^ percentile of expression values was excluded from the analysis. For genes that had multiple entries in the gene list, only the list entry with the highest expression in control samples was used for further analysis.

Expression values with an absolute fold change (FC) ≥ 1.3 between control and *Cstb*^*-/-*^ were considered differentially expressed. Statistical testing was done using T-test with unpaired unequal variance corrected with Benjamini-Hochberg multiple testing correction and an adjusted p-value < 0.05 was considered significant. The microarray gene expression data are accessible in the Gene Expression Omnibus (GEO, NCBI, www.ncbi.nlm.nih.gov/geo/) repository (ID: GSE64823).

### Transcriptome analysis by RNA-seq

Bulk-RNA transcriptome analysis of four technical replicates per each of the five *Cstb*^*-/-*^ and control microglial RNA samples was performed by the RNA-seq method, followed by the single-cell tagged reverse transcription (STRT) [22] protocol with modifications [23]. 10 ng of total RNA was converted to cDNA and amplified to form an Illumina-compatible library. Artificial ERCC RNA Spike-In Mix 1 (Life Technologies, USA) was diluted to 1:1000 and 1 μl was used to enable data normalization. In total, 25 PCR cycles were used, but as four base-pair unique molecular identifiers were applied, only the absolute number of unique reads was calculated per analysed sample. The library was sequenced on one lane of Illumina HiSeq2000, further processed to fastq files by Casava 1.8.2 (both Illumina, San Diego, CA, USA), and quality control was performed using the STRTprep pipeline (https://github.com/shka/STRTprep) [23]. Briefly, the filtered sequence reads were aligned to the mouse UCSC genome mm9 [24], the mouse ribosomal DNA repetitive unit (GenBank: BK000964), the *E*. *coli* ynbA (GenBank: EF011072 as negative control), and the ERCC spike-in RNAs by TopHat [25] with UCSC Known Genes (version from 23.06.15) [26] as transcriptome reference. In the data analysis, we excluded the same two RNA samples (one *Cstb*^*-/-*^ and one control sample) that were excluded in the microarray analysis. The raw read frequencies are counts of the STRT reads, which aligned within the 5’-UTR or up to 500 bp upstream of each protein-coding gene in the sample. The normalized expression level is the raw read frequency divided by total read counts, which aligned to the ERCC spike-ins in the sample, as spike-in normalization. From all identified genes, a gene was considered expressed if more than two of the normalized expression values from all the technical repeats of the control and *Cstb*^*-/-*^ samples were greater than zero. The genes differentially expressed between control and *Cstb*^*-/-*^ RNA samples were identified by SAMstrt, with spike-in based normalization [27] and by their degree of fluctuation or variation [23]. We considered genes with a FDR q-value < 0.01 and a significant fluctuation (adjusted p-value < 0.01) as differentially expressed between control and *Cstb*^*-/-*^ samples.

### Platform comparison

The platform comparison was performed between the expression data obtained by microarray and RNA-seq approach. The microarray gene expression values summarized by RMA16 were filtered for protein-coding genes using the current, protein-coding mouse (taxon ID: 10090) genes from NCBI Gene (version from 13.11.15) [28] and were compared to the normalized gene expression values obtained by RNA-seq. Correlation was determined by Spearman’s rank correlation coefficient.

### Functional annotation of the transcriptome

Gene ontology (GO) terms enriched in the differentially expressed genes in *Cstb*^*-/-*^ microglia were identified using the web-based gene ontology enrichment analysis and visualization tool GOrilla (www.cbl-gorilla.cs.technicon.ac.il, version from 22.07.2015) [29, 30]. A p-value threshold of 0.001 was considered significant. Canonical pathways and upstream regulators enriched in the differentially expressed genes were identified using QIAGEN´s Ingenuity Pathway Analysis (IPA, QIAGEN, Redwood City, US, www.qiagen.com/ingenuity, version from 22.07.15) in a Core analysis using the Fisher´s Exact test and a p-value < 0.05 cutoff. In the core analysis, identified upstream regulators were ranked by a z-score, which reflected the probability that the regulator underlay the observed expression changes. The protein network was generated with the protein-protein interaction database Search Tool for the Retrieval of Interacting Genes/Proteins (STRING) 10 (www.string-db.org, version from 18.09.2015) [31] and illustrated with Cytoscape 3.2 [32].

### Quantitative real-time PCR (qPCR)

Isolation of total RNA from cultured control and *Cstb*^*-/-*^ mouse microglia was performed using the RNeasy Mini kit (Qiagen, Hilden, Germany) according to the manufacturer’s instructions. For each sample, the RNA of 2–4 mice per genotype was pooled. The concentration and purity of the RNA was assessed spectrophotometrically (ND-1000, NanoDrop Technologies, Wilmington, DE) and RNA was transcribed to cDNA using the iScript cDNA Synthesis Kit (Bio-Rad Laboratories, Hercules, CA) according to the manufacturer’s instructions. Absence of genomic DNA in the RNA samples was determined by amplification of the ribosomal protein S15 by PCR. TaqMan Gene Expression assays (Applied Biosystems, Foster City, CA) were used for quantitative real-time PCR (qPCR), which was performed on an ABI Prism 7000 Sequence Detection System according to the manufacturer’s instructions. These TaqMan Gene Expression Assays were used: *mStat1* (Mm00439531_m1), *mIrf7* (Mm00516793_g1), *mIrf9* (Mm00492679_m1), and *mTbp* (Mm00446973_m1). DataAssist software Version 3.01 (Applied Biosystems) was used to perform relative quantification of the data using the comparative Ct (ddCt) method [33]. The Tata-binding protein (*Tbp*) transcript expression was used as endogenous control and statistical tests were performed using GraphPad Prism version 6.02 for Windows (GraphPad Software, La Jolla, California, USA, www.graphpad.com). Statistical significance between control and *Cstb*^*-/-*^ samples was determined using the unpaired t-test with Welch’s correction and a significance level of p < 0.05 was considered statistically significant.

### JAK-STAT signaling pathway PCR assay

Total RNA was extracted from primary cultured microglia cells from control and *Cstb*^*-/-*^ mice using the RNeasy Mini kit (Qiagen, Hilden, Germany) according to the manufacturer’s instructions. For each sample, the RNA of 3 mice per genotype was pooled. RNA concentration and purity was determined spectrophotometrically (ND-1000) and the mouse JAK/STAT Signaling Pathway RT^2^ Profiler PCR Array (Qiagen) was performed according to the manufacturer’s instructions using the ABI Prism 7000 Sequence Detection System (Applied Biosystems).

## Results

### The majority of differentially expressed genes in *Cstb*^*-/-*^ microglia are downregulated

To generate a comprehensive transcript profile of CSTB-deficient microglia, we assessed gene expression changes between *Cstb*^*-/-*^ and control microglia with two different expression profiling methods, microarray analysis and RNA-seq.

First, we analyzed the microglia RNA samples by microarray using the Affymetrix Mouse Exon 1.0 ST arrays and identified 14 147 expressed transcript clusters. To pinpoint the relevant expression changes in *Cstb*^*-/-*^ microglia, we then focused on the transcript clusters with an absolute FC of at least 1.3 and a corrected p < 0.05, and detected 155 differentially expressed genes (DEGs; S1 Table). Of these, only four genes were upregulated (FC range: 1.3 to 1.8; highest FC: lysophosphatidylcholine acetyltransferase 4 (*Lpcat4*)), and the majority, 151 genes, were downregulated (FC range: -9.3 to -1.3). The downregulated genes included several interferon-regulated genes, such as interferon-induced protein with tetratricopeptide repeats 1 (*Ifit1*, FC: -8.1), 2’-5’ oligoadenylate synthetase-like 2 (*Oasl2*, FC: -6.6), and interferon regulatory factor 7 (*Irf7*, FC: -5.3).

Second, we performed RNA-seq of the microglia RNA samples using the single-cell tagged reverse transcription (STRT) protocol [22]. After removal of redundant reads, which are generated during PCR amplification, we obtained on average 2 129 122 (± 7.1%) total reads per sample sequenced on one Illumina HiSeq2000 lane. Of these total reads, 1 884 590 (± 7%) per sample were mapped to the genome (mapped rate), 1 654 282 (± 7.1%) per sample were mapped to coding sequence and 1 337 991 (± 7.5%) per sample were mapped to coding 5’ regions (S2 Table). In this analysis, we detected 11 222 genes with altered expression in *Cstb*^*-/-*^ microglia based on the variation in the number of sequence reads obtained from added RNA spike-ins by SAMstrt [27], which has been utilized to assess differential expression previously [34, 35]. To focus on the significant differences, we considered only genes with a false discovery rate (FDR) < 0.01 and a significant fluctuation (adjusted p-value < 0.01), and 62 genes passed this criteria (S1 Table). Of these, four genes were upregulated (FC range: 1.8 to 4.8; highest FC: serine peptidase inhibitor, clade B, member 10 (*Serpinb10-ps*)), and 58 genes were downregulated (FC range: -200 to -2.2). Among the most downregulated genes, a few were interferon-regulated genes, e.g. *Oasl2* (FC: -17.9), ISG15 ubiquitin-like modifier (*Isg15*) (FC: -11.9), *Ifit3* (FC: -11.8), and *Ifit1* (FC: -11.2).

### Microarray and RNA-seq results are concordant

To investigate the *Cstb*^*-/-*^ microglia transcriptome, we examined the expression data in more detail. In this analysis, we restricted the genes detected by microarray to protein-coding genes, because the standard procedure of STRTprep for differential expression and pathway analysis specifically targets protein-coding genes.

**Fig 1:**
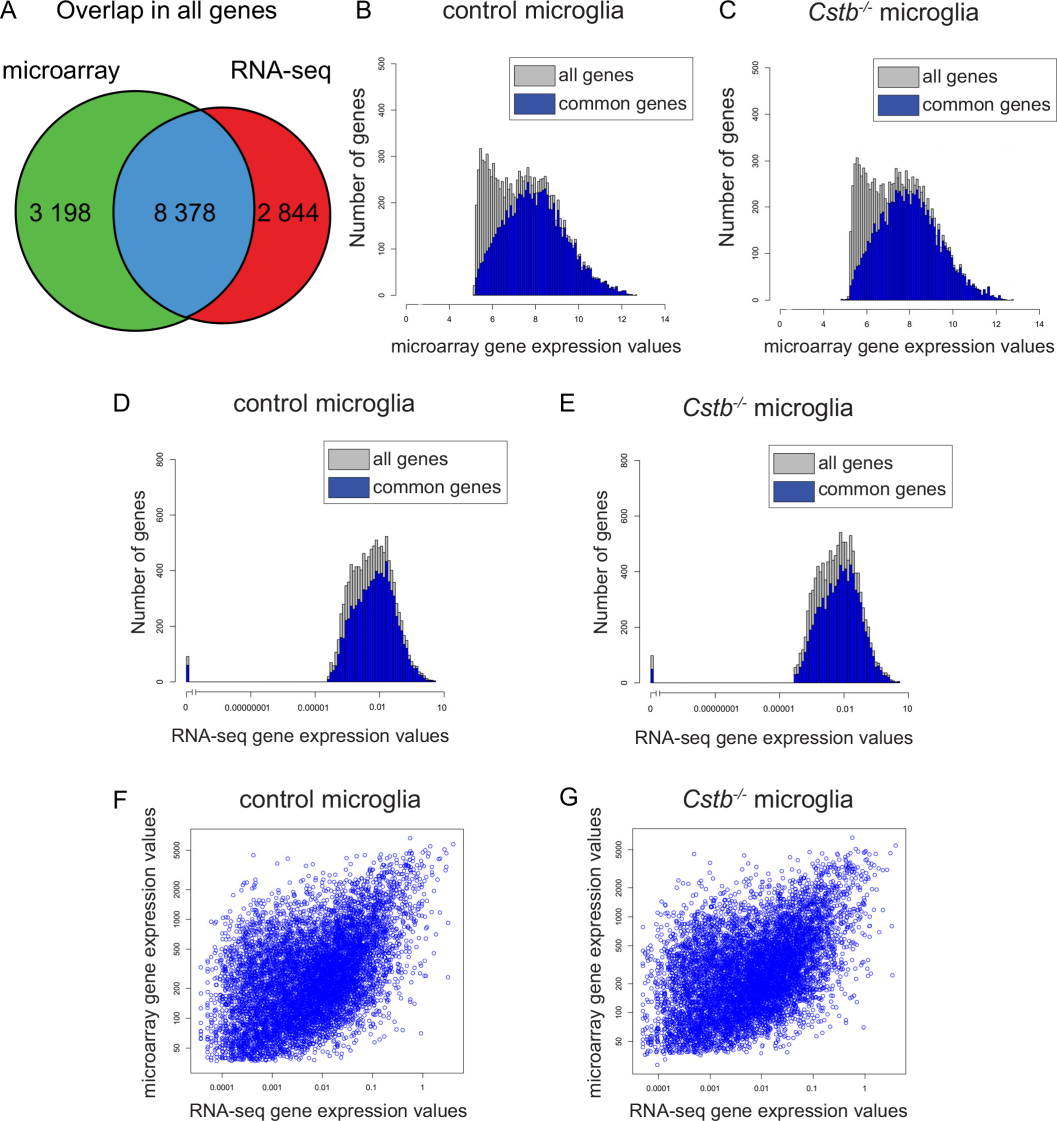
Gene expression values identified by microarray- and sequencing-based transcriptome profiling of *Cstb*^*-/-*^ microglia. (A) Venn diagram illustrating the overlap in the number of genes identified by both methods (blue), only by microarray (green), or only by RNA-seq (red). (B–E) Distribution of microarray and RNA-seq gene expression values in control and *Cstb*^*-/-*^ microglia. The expression values of genes identified also in the other method (blue bars) are higher than the expression values identified only by the microarray or the RNA-seq (grey bars). The number of genes with a specific gene expression value is depicted for (B) control and (C) *Cstb*^*-/-*^ microglia in the microarray and for (D) control and (E) *Cstb*^*-/-*^ microglia in the RNA-seq data. Scatter plot of the mean expression values for each gene of (F) control and (G) *Cstb*^*-/-*^ samples identified by both methods.

Overall, the total transcription profiling of *Cstb*^*-/-*^ microglia provided expression data of 14 420 genes. Of these, 2 844 (20%) were identified only by RNA-seq, 3 198 (22%) were uniquely identified by microarray, and 8 378 (58%) were detected by both techniques (Fig 1A). Interestingly, genes that were identified to be differentially expressed only by one of the methods showed weaker expression than genes identified by both techniques (Fig 1B–1E). To explore the consistency between the microarray and the RNA-seq data, we determined the accordance between gene expression values, and found a moderate correlation both in control (Spearman´s rank correlation coefficient, SCC: 0.47) and in *Cstb*^*-/-*^ microglia (SCC: 0.45) (Fig 1F and 1G). To evaluate this further, we investigated, as a measure less dependent on the normalization method, also the correlation between the fold changes obtained by microarray and by RNA-seq (SCC: 0.51), which was comparable to the correlation between expression values.

**Fig 2:**
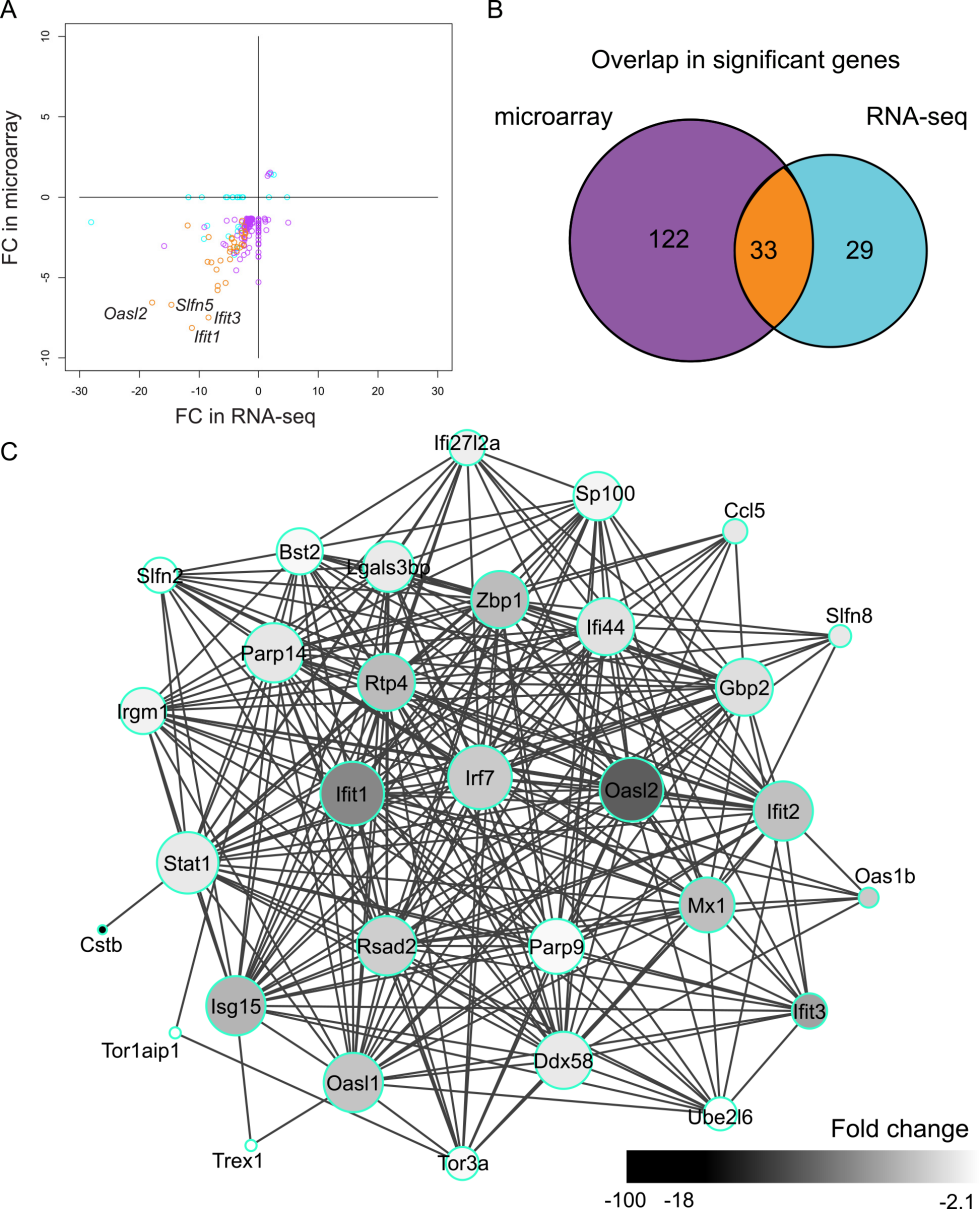
DEGs identified by microarray- and sequencing-based expression profiling of *Cstb*^*-/-*^ microglia. (A) Scatter plot of the FCs of the differentially expressed genes (DEGs) identified by microarray and RNA-seq. DEGs common to both methods are depicted in orange, RNA-seq-specific DEGs in light blue, and microarray-specific DEGs in purple. (B) Venn diagram showing the overlap in the number of DEGs. The colors correspond to the colors in A. (C) Protein-protein interaction network of the 33 genes differentially expressed in microarray and RNA-seq. The size of each node is proportional to the number of its connections to other nodes and the color of each node illustrates the fold change of the gene it represents.

Altogether, 184 genes are differentially expressed in *Cstb*^*-/-*^ microglia (S1 Table). We observed good accordance in DEGs identified by both methods, which was expected as the number of polyadenylated transcripts in control and *Cstb*^*-/-*^ RNA samples was similar (589 and 598 for 10 ng RNA, respectively). In addition, we observed good correlation between the fold changes of these genes (SCC: 0.73) (Fig 2A). Only six DEGs (3.2%) had different fold change directions. They were altered only in the microarray-based data, and were therefore probably false positive discoveries. Of the 184 DEGs in *Cstb*^*-/-*^ microglia, 33 (18%) genes were shared by both methods (marked with an asterisk (*) in S1 Table) (Fig 2B). Although their number was small, the overlap was significantly higher than expected (odds ratio = 132.2, p < 2.2e-16 by Fisher’s Exact test for Count Data, S3 Table), and their gene expression levels were not lower than the levels of the non-overlapping DEGs (S1 Fig). They showed good accordance in their fold changes (SCC: 0.75) and formed a highly interconnected network (Fig 2C), in which for example *Irf7*, *Ifit1*, *Oasl2*, and *Stat1* had central positions. In addition, 122 DEGs were altered uniquely in the microarray profiling. Of these, 98 (80%) were identified, but not altered, in the RNA-seq profiling. Moreover, 29 genes were changed only in the RNA-seq data and of these 13 (45%) were detected, but not altered, in the microarray profiling.

In summary, the analysis demonstrated good concordance between both technologies and provided a detailed and solid signature of CSTB deficiency in microglia.

### RNA expression profiles indicate that microglia are neither pro- nor anti-inflammatory polarized

**Fig 3:**
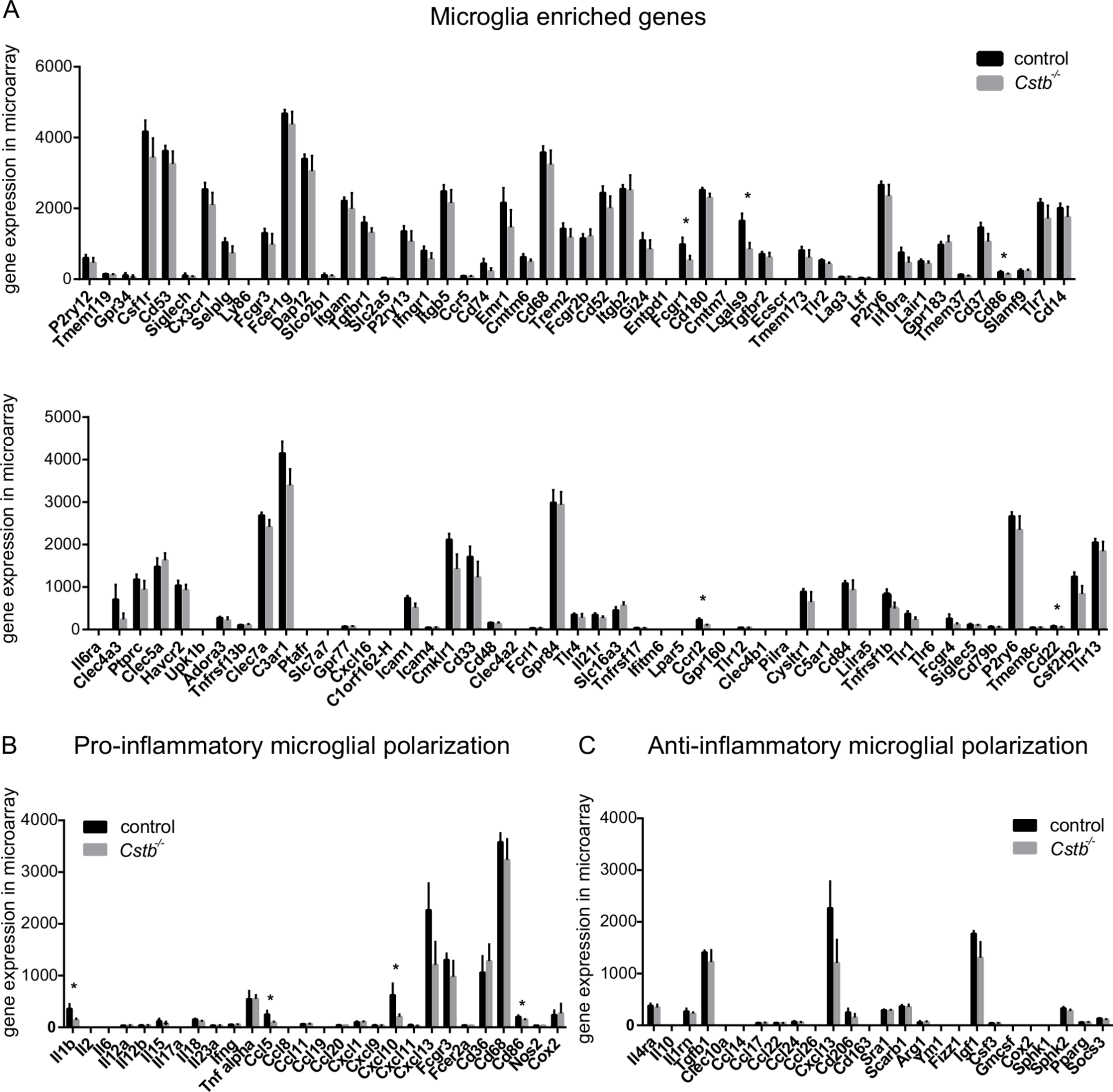
Microarray expression values of genes associated with microglia or microglial activation in control and *Cstb*^*-/-*^ microglia. Microarray gene expression level of transcripts (A) reportedly enriched in microglia, (B) associated with pro-inflammatory, and (C) with anti-inflammatory activation of microglia determined in control (black bars) and *Cstb*^*-/-*^ (grey bars) microglia. The asterisk (*) marks genes significantly altered in *Cstb*^*-/-*^ microglia compared to control microglia. Error bars represent standard deviation (SD).

In order to assess how microglia-specific the transcription profiles of the control and *Cstb*^*-/-*^ samples were compared to known microglial transcript expression profiles, we extracted from the microarray and the RNA-seq data the expression values of 100 genes that have been reported to be highly expressed in microglia [36]. In control and *Cstb*^*-/-*^ microglia, 81 of these genes were detected by microarray (Fig 3A) and 93 by RNA-seq profiling (S2A Fig). Of these genes, Fc gamma receptor 1 (*Fcgr1*), lectin, galactose binding, soluble 9 (*Lgals9*), CD86 antigen (*Cd86*), CD22 antigen (*Cd22*), and chemokine (C-C-motif) receptor-like 2 (*Ccrl2*) were downregulated in *Cstb*^*-/-*^ microglia in the microarray data (marked with an asterisk in Fig 3A) and C-type lectin domain family 4, member a3 (*Clec4a3*) in the RNA-seq data (marked with an asterisk in S2A Fig).

In general, microglia are very sensitive to disturbances, and react with morphological and gene expression changes to alterations in their environment. We therefore also evaluated the expression of markers for activation of control and *Cstb*^*-/-*^ microglia in the microarray and RNA-seq data. To achieve this, we focused on the expression levels of transcripts previously associated with pro-inflammatory or anti-inflammatory microglial polarization [37]. In control and *Cstb*^*-/-*^ microglia, genes linked to pro- and anti-inflammatory function were expressed, but the microglia were not polarized to either state (Fig 3B and 3C). The expression of only four pro-inflammatory genes, namely *Cd86*, Interleukin 1β (*Il1b*), and chemokine (C-C motif) ligand 5 (*Ccl5*) and chemokine (C-X-C motif) ligand 10 (*Cxcl10*), was significantly altered between *Cstb*^*-/-*^ and control microglia (marked with an asterisk in Fig 2B) in the microarray and *Ccl5* and *Cxcl13* in the RNA-seq data (marked with an asterisk (*) in S2B and S2C Fig).

In conclusion, these data suggest that CSTB deficiency did not substantially alter the expression of genes that were considered central for the transcriptional signature defining microglia cells or for the regulation of microglial activation.

### Functional enrichment analysis indicates impaired immune-system related functions in *Cstb*^*-/-*^ microglia

To explore more deeply the biological context of the DEGs in *Cstb*^*-/-*^ microglia, we determined their associated biological processes, molecular functions, and cellular locations using Gene Ontology (GO) enrichment analysis, which we performed separately for the microarray (S4 Table) and the RNA-seq data (S5 Table).

The most significantly enriched *biological processes* in *Cstb*^*-/-*^ microglia in both analyses included GO terms associated with immune and defense response, as well as interferon (INF) signaling. In addition, the *Cstb*^*-/-*^ microglia DEGs were implicated in processing and presentation of antigens. This category comprised genes that are involved in generation of antigenic peptides in the proteasome (proteasome subunit beta type-9 (*Psmb9*)) and in peptide translocation from cytoplasm into the endoplasmic reticulum (transporter 1, ATP-binding cassette, sub-family B (MDR/TAP) (*Tap1*) and Tap binding protein (*Tapbp*)). Furthermore, we identified GO terms linked to migration and chemotaxis exclusively in the RNA-seq results. This category included e.g. the cytokines *Cxcl3*, *Il16*, and *Ccl2*, as well as the complement receptors integrin alpha M (*Itgam*) and complement component 5a receptor 1 (*C5ar1*).

The *molecular functions* enriched in *Cstb*^*-/-*^ microglia included nucleotide binding, GTPase activity as well as chemotaxis. The microarray DEGs were involved in nucleotide binding, which comprised several oligoadenylate synthases (Oas) (*Oas1a/g*, *Oas1b*, and *Oas2*) that synthesize 2´-5´-linked oligoadenylates in an ATP-dependent manner, as well as the OAS-like proteins 1 and 2 (*Oasl1*, *Oasl2*). In addition, this category contained genes encoding schlafen (SLFN) proteins (*Slfn2*, *Slfn5*, *Slfn8/9*) and several helicases (DEXH box polypeptide 58 (*Dhx58*), activating signal cointegrator 1 complex subunit 3 (*Ascc3*), DEAD box polypeptide 58 (*Ddx58*), interferon induced with helicase C domain 1 (*Ifih1*), and Moloney leukemia virus 10 (*Mov10*)). The genes possessing GTPase activity were interferon-inducible GTPases. This category included genes such as Mx dynamin-like GTPase 1 (*Mx1*), *Mx2*, guanylate binding protein 2/1 (*Gbp2/1*), *Gbp6/3*, immunity-related GTPase family M member 1 (*Irgm1*), *Irgm2*, and very large interferon inducible GTPase 1 (*Gvin1*). The RNA-seq-based molecular functions highlighted the association of CSTB deficiency with chemotaxis, including genes such as *Ccl2*, *Cxcl13*, *Cxcl2*, *Cxcl3*, and *Cxcl10*.

The GO terms describing *cellular components* in *Cstb*^*-/-*^ microglia were associated with cell membranes and the extracellular space in both methods.

### Interferon signaling pathway is downregulated in *Cstb*^*-/-*^ microglia

**Fig 4:**
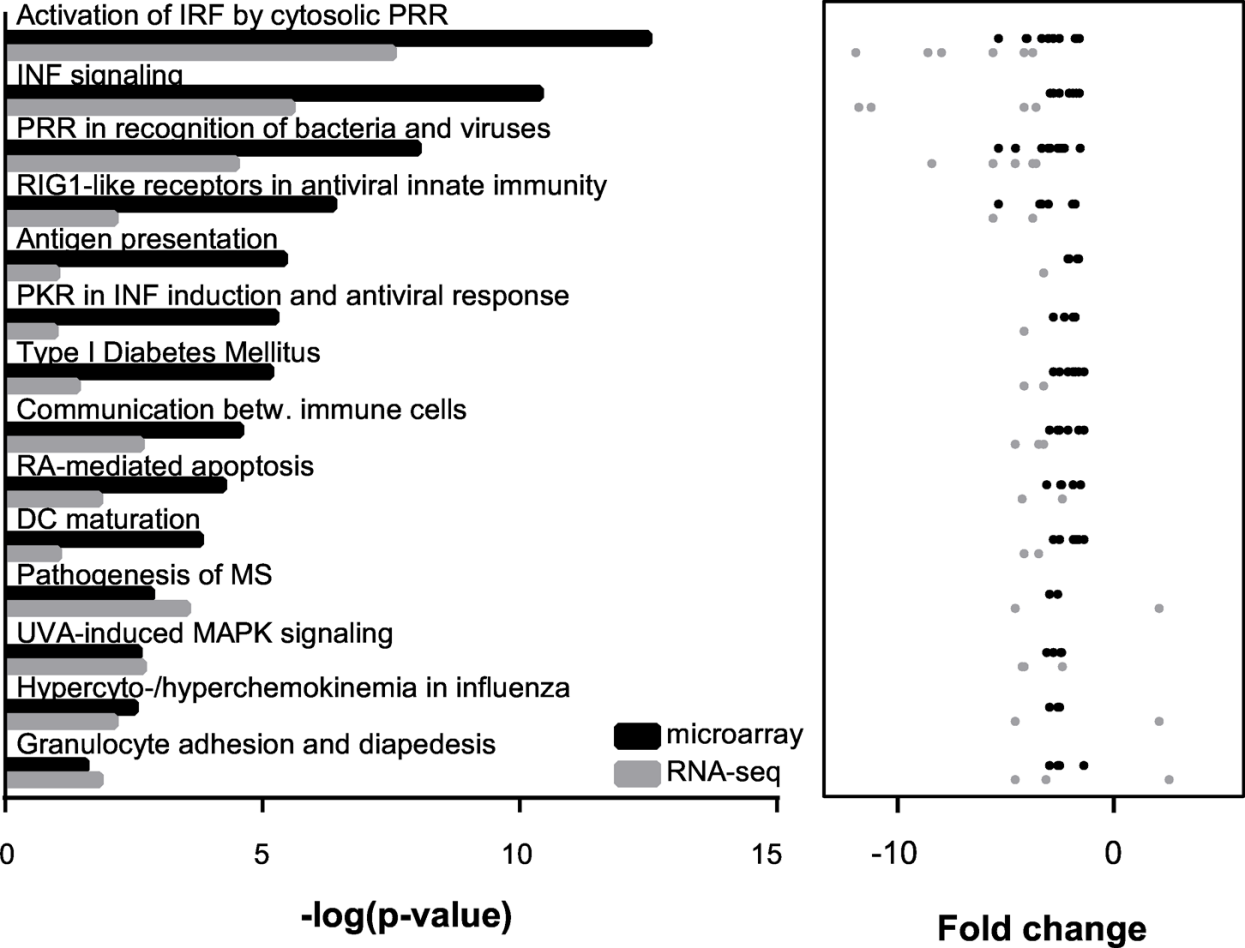
Canonical pathways enriched in *Cstb*^*-/-*^ microglia identified by microarray and RNA-seq analyses. The 14 canonical pathways most highly ranked based on their p-value in the microarray (black color) and the RNA-seq approach (grey color) are depicted. The p-values and the fold changes of the microglia genes linked to each pathway are illustrated for both methods results.

**Fig 5:**
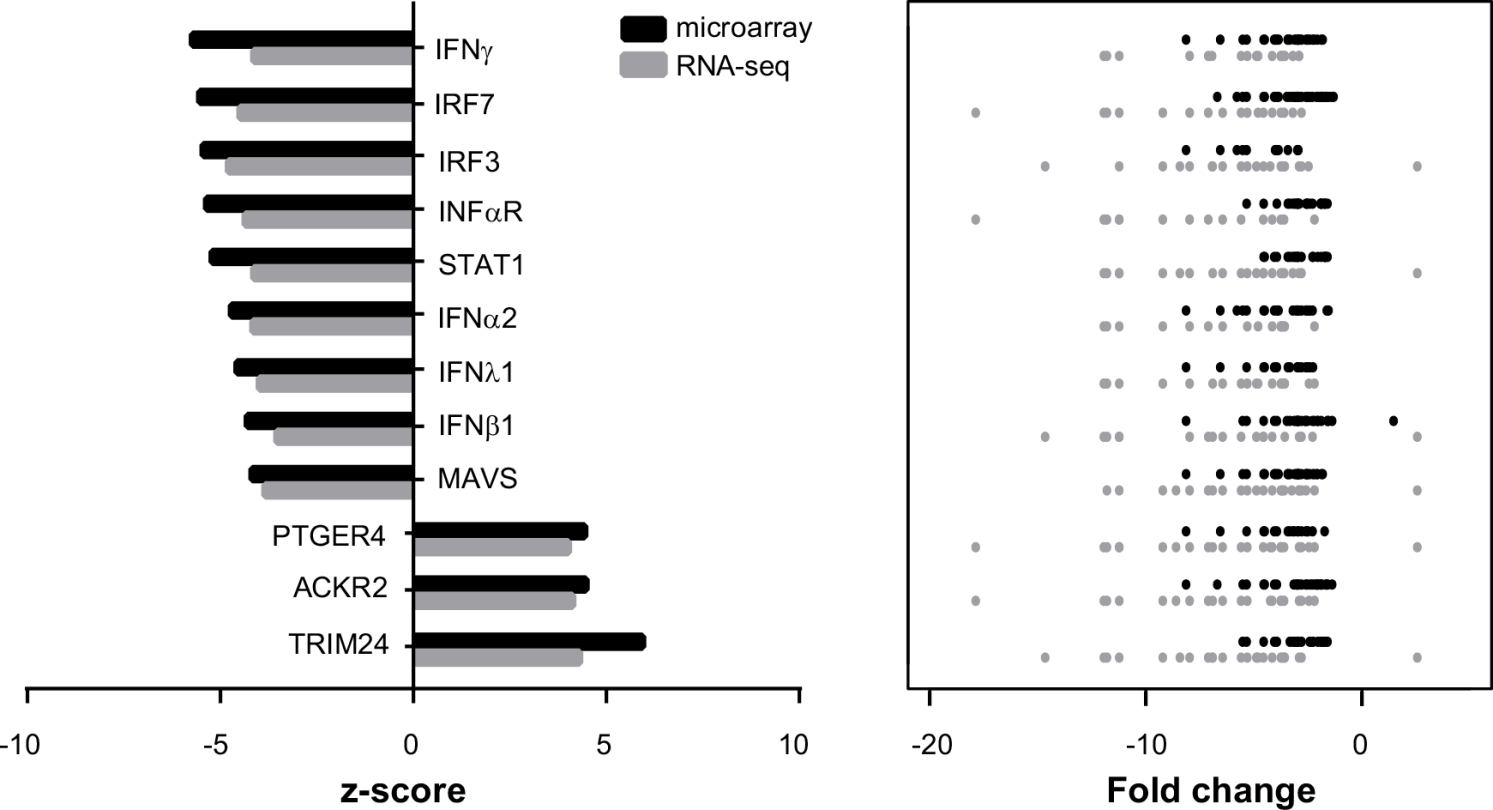
Upstream regulators enriched in *Cstb*^*-/-*^ microglia identified by microarray and RNA-seq analyses. The 12 upstream regulators most highly ranked based on their p-value in the microarray (black color) and the RNA-seq approach (grey color) are depicted. The z-scores and the fold changes of all genes potentially controlled by this regulator are illustrated for both methods.

Next, we asked whether the DEGs shared common regulators potentially mediating the gene expression changes in *Cstb*^*-/-*^ microglia and if these genes were part of the same biological pathway. Therefore, we examined the microarray and the RNA-seq findings for molecular pathways (Fig 4, detailed results in S6 Table) and potential upstream regulators (Fig 5, detailed results in S7 Table) linked to CSTB deficiency in microglia.

The molecular pathways over-represented in *Cstb*^*-/-*^ microglia in both methods were involved in regulation of the innate immune system and interferon signaling (Fig 4). They included for example the recognition of pathogens and cellular damage by pattern recognition receptors (PRRs), the activation of interferon-regulated factors (IRFs), and signaling via the interferon pathway. The upstream regulators enriched in *Cstb*^*-/-*^ microglia comprised the mitochondrial antiviral signaling protein (MAVS), the transcription factors IRF7, IRF3, and Signal transducer and activator of transcription 1 (STAT1), as well as several interferons (IFNλ1, IFNα2, IFNβ1, and IFNɣ), and the interferon-α/β receptor (INFαR) (Fig 5). The majority of these regulators act either upstream of the JAK-STAT signaling pathway or control the signal transduction of this pathway [38], suggesting that it might mediate the altered expression of interferon regulated genes in *Cstb*^*-/-*^ microglia.

To further investigate this, we performed qPCR of interferon-pathway regulators *Irf7*, *Irf9*, and the transcription factor *Stat1* and a qPCR-based JAK-STAT pathway assay from control and *Cstb*^*-/-*^ microglia RNA samples. The qPCR confirmed the downregulation of *Irf7* and *Stat1* (S3 Fig). However, expression of the interferon-regulated gene *Irf9*, which was only slightly downregulated on the microarray (FC: -1.6), was reduced, but did not reach statistical significance. The JAK-STAT pathway assay, which determines transcript levels of 84 genes previously associated with this pathway, revealed only modest gene expression changes in *Cstb*^*-/-*^ microglia. We utilized a FC cut off of 1.3 and identified four downregulated genes (FC range: -1.3 to -1.5) and 40 upregulated genes (FC: 1.3 to 2.5) (Table 1). Using this assay, we were able to pinpoint the downregulation of *Irf9*.

**Table 1:** JAK-STAT pathway-associated transcripts differentially expressed in *Cstb*^*-/-*^ microglia.

Gene Symbol	FC	Gene Symbol	FC	Gene Symbol	FC
*Irf9*	-1.5	*Il20*	1.5	*Socs5*	1.7
*Smad2*	-1.4	*Nr3c1*	1.5	*Jak3*	1.7
*Sp1*	-1.4	*Il10rb*	1.5	*Myc*	1.7
*Csf1r*	-1.3	*Il4*	1.5	*Cxcl9*	1.7
*Il10ra*	1.3	*Fcer2a*	1.5	*F2*	1.8
*Sh2b2*	1.3	*Junb*	1.5	*Lrg1*	1.8
*Pdgfra*	1.3	*Il4ra*	1.5	*Nos2*	1.8
*Pias1*	1.3	*Ifng*	1.5	*Socs2*	1.9
*Ifngr1*	1.3	*Hmga1*	1.6	*Prl*	2.0
*Ptpn1*	1.3	*Osm*	1.6	*Jun*	2.1
*Socs3*	1.4	*A2m*	1.6	*Mpl (TPO-R)*	2.1
*Crp*	1.4	*Stat5a*	1.6	*Epor*	2.1
*Bcl2l1*	1.4	*Tyk2*	1.6	*Gata3*	2.4
*Socs1*	1.4	*Ghr*	1.6	*Stat4*	2.5
*Smad4*	1.5	*Src*	1.7		

Relative gene expression levels were determined by JAK-STAT pathway array and the fold change (FC) between control and *Cstb*^*-/-*^ microglia was calculated. All genes with an absolute FC ≥ 1.3 are considered differentially expressed.

In summary, our results showed a reduced expression of interferon-regulated genes in *Cstb*^*-/-*^ microglia and implied a putative role for CSTB in regulation of interferon signaling.

## Discussion

Our previous findings of an early activation and dysfunction of *Cstb*^*-/-*^ microglia with consequent neuroinflammation [14, 19] and an upregulation of genes linked to immune system processes in cerebella of one-month-old *Cstb*^*-/-*^ mice [18] strongly supported a contribution of microglial dysfunction and inflammatory processes in the pathogenesis of EPM1. To scrutinize especially the microglial dysfunction associated with CSTB deficiency in more depth, we here focused on global gene expression profiling of cultured *Cstb*^*-/-*^ and control microglia by applying a combination of two widely used gene expression profiling methods, microarray and RNA-seq, to the same RNA samples. Transcriptome profiling is a useful method to pinpoint altered cellular pathways. For example in neural stem cells, Notch signaling has been shown to be regulated by sirtuin 1(SIRT1) using this technique [39]. In *Cstb*^*-/-*^ mice, we have successfully utilized this method previously and found altered expression of synaptic genes in the cerebellum, which led to the finding of reduced GABAergic inhibition of *Cstb*^*-/-*^ cerebellar Purkinje cells [18]. Our comparison of the *Cstb*^*-/-*^ microglia microarray and RNA-seq results revealed good concordance between both techniques, which was expected due to the similarities in the amounts of polyadenylated transcripts in *Cstb*^*-/-*^ and control samples. For example, more than half of all identified genes were detected by both methods, and their gene expression values as well as fold changes correlated reasonably well. The correlation in gene expression values was slightly less strong, but comparable with earlier reports comparing RNA-seq and microarrays [40–42]. Moreover, we found good concordance between the functional enrichment analyses, although the number of DEGs shared by both methods was small. This might be due to the different normalization methods used in both studies. The RNA-seq data are normalized to spike-in RNAs with known sequence and concentration that are added to the samples and the microarray data are normalized between arrays by quantile normalization, although GeneChips can also be normalized by spike-in RNAs, e.g. [43]. In addition, the probe selection on the microarray might underlie the differences. The microarray probes are spread over different exons in the genes, whereas the STRT RNA-seq protocol only counts mapped reads within the 5’-UTR or up to 500 bp upstream of known protein-coding genes. Moreover, the STRT RNA-seq detects only intact mRNAs, which are used as templates for protein translation, but it is not ideal for the detection of some longer mRNAs, because it uses oligo-dT primer for the first-strand cDNA synthesis. In contrast, the microarray uses random primer and therefore measures degrading mRNAs as well, but it is better for long mRNAs. Sequencing depth for the RNA-seq analysis is another factor, the optimal sequencing depth depends on the aim of the study and on the transcriptome complexity of the target sample. In addition, the amount of input RNA influences the detection power. In this first STRT study, we used 10 ng total RNA for each replica (equivalent to 40 ng for each sample; while 100 ng for the microarray), and sequenced one STRT library containing all replicas of all samples on one HiSeq2000 lane. Although this setup identified dominant gene expression changes, which were also detected by microarray, a much deeper sequencing might also reveal more subtle expression changes [44]. Although there were many possibilities, one consistent result was that in both approaches the majority of genes showing significantly altered expression in *Cstb*^*-/-*^ microglia were downregulated.

The adult mouse microglia transcriptome has been determined by RNA-seq [36] and the 100 genes with highest expression were linked to the detection of molecules, such as pattern recognition receptors, and purinergic receptors, and thus have been called the microglial ‘sensome’. The evaluation of the sensome gene expression in *Cstb*^*-/-*^ and control microglia revealed that the majority of these genes are also expressed in primary microglia, although both studies differed in extraction and culturing of the cells. In addition, the expression of microglial sensome genes was not altered considerably in *Cstb*^*-/-*^ microglia compared to control microglia. To characterize not only the sensome of *Cstb*^*-/-*^ microglia, but also their activation, the expression of pro- and anti-inflammatory phenotypic markers can be analyzed [37]. Our previous study showed that in *Cstb*^*-/-*^ mice, the balance between pro-inflammatory and anti-inflammatory activated microglia was, compared to controls, shifted towards anti-inflammatory type at P14 and towards pro-inflammatory type in P30. We also described upregulated protein expression of both the pro-inflammatory marker iNOS and the anti-inflammatory marker Arginase1 in P30 *Cstb*^*-/-*^ mouse brain [19]. However, the primary cultured *Cstb*^*-/-*^ microglia in this study were neither pro- nor anti-inflammatory activated *in vitro*. We noted a down-regulation of five pro-inflammatory genes (*Cd86*, *Il1b*, *Ccl5*, *Cxcl10*, *Cxcl13*), which, however, might be due to the altered interferon signaling, because most of these genes were interferon regulated according to the database Interferome 2.0 [45].

The GO enrichment analysis indicated that CSTB deficiency in microglia altered immune system- and defense response-related functions. More specifically, GO terms associated with antigen presentation and chemotaxis were over-represented in *Cstb*^*-/-*^ microglia. These data are in line with previous *in vitro* studies in *Cstb*^*-/-*^ microglia and bone marrow-derived macrophages (BMDM), which have shown altered immune cell functions, suggesting e.g. changes in antigen presentation and chemotaxis [11, 19, 46]. Further analyses of the altered pathways and their upstream regulators indicated that the mechanism underlying the dysfunction of *Cstb*^*-/-*^ microglia might be associated with interferon signaling and the JAK-STAT pathway. Interferon signaling is an important regulator of the innate immune response mediating the defense against viral infections [38]. However, in recent years also other functions for interferon signaling have been emerging, e.g. the regulation of autophagy [47]. Both enhanced and impaired interferon signaling has been linked to pathological conditions [48]. For example, enhanced IFN signaling has been associated with autoimmune diseases, such as rheumatoid arthritis and systemic lupus erythematosus [49]. In contrast, IFNβ-mediated signaling ameliorated pathology in ischemic stroke and cerebral ischemia [50, 51], suggesting a beneficial role for interferon signaling. Moreover, STAT1 and IRF9 are protective against neuropathology mediated by chronic astrocytic IFNα expression, which induces e.g. neurodegeneration, inflammation, and glial activation [52, 53]. Our results indicating altered interferon regulated JAK-STAT signaling in microglia are in line with previous studies analyzing *Cstb*^*-/-*^ BMDMs of adult mice. These showed reduced activation or expression of proteins on this pathway in response to pro-inflammatory stimulation, e.g. reduced STAT1 activation by tyrosine phosphorylation and *Ifnb1* mRNA expression [11, 46]. In *Cstb*^*-/-*^ microglia, *Stat1* mRNA abundance was reduced, however, *Ifnb1* expression was not altered, which might be due to a cell type specific downregulation of *Ifnb1* in *Cstb*^*-/-*^ BMDMs. In addition, we might not be able to detect the downregulation of *Ifnb1*, because of its weak constitutive expression level [54].

Reduced signaling via the interferon pathway in *Cstb*^*-/-*^ microglia might also have implications for other cellular processes. For example, IFNβ-induced IFNAR signaling inhibits reactive oxygen species production and inflammasome activation [55]. Thus, reduced IFN signaling in *Cstb*^*-/-*^ mice might elevate inflammasome activation and induce the activation of the pro-inflammatory cytokines IL1β and IL18, which have been shown at high levels in the serum of LPS-injected *Cstb*^*-/-*^ mice [11] and have been linked to the pathogenesis of several neurodegenerative diseases, such as Alzheimer’s disease and multiple sclerosis [56]. We observed a decreased *Il1b* mRNA expression in *Cstb*^*-/-*^ microglia, however, the microglia samples in our study were not pro-inflammatory activated.

In addition to affecting the immunological functions, altered interferon signaling in *Cstb*^*-/-*^ immune cells might have a more direct impact on neuronal signaling and/or viability. For example, IFNγ has been reported to suppress the differentiation of GABAergic neurons from neuronal progenitor cells, and to induce the generation of glutamatergic neurons [57]. Although we were not able to pinpoint altered *Ifng* expression in *Cstb*^*-/-*^ microglia, it is, in light of our previous electrophysiological recordings of P7 *Cstb*^*-/-*^ Purkinje cells, which have revealed an imbalance between excitation and inhibition in *Cstb*^*-/-*^ mouse brain [18], tempting to speculate that impaired interferon signaling in *Cstb*^*-/-*^ microglia could modify the excitability and viability of *Cstb*^*-/-*^ neurons.

## Conclusion

Collectively, the integration of data from microarray and RNA-seq technologies strengthened and expanded the previously reported indications of impaired interferon-signaling pathway in *Cstb*^*-/-*^ mice to a general downregulation of interferon-regulated genes [11, 46]. Moreover, our results supported previous findings suggesting that modified inflammatory processes of microglia contribute to the pathogenesis of EPM1.

## Supporting Information

S1 Fig DEG expression level. The DEG expression level in (A) microarray and (B) RNA-seq. Microarray specific DEGs are depicted in violet, RNA-seq specific DEGs in light blue, and DEGs common to both methods in orange.
(PDF)

S2 Fig RNA-seq expression values of genes associated with microglia or microglial activation in control and *Cstb*^*-/-*^ microglia. RNA-seq gene expression level of transcripts (A) reportedly enriched in microglia, (B) associated with pro-inflammatory, and (C) with anti-inflammatory activation of microglia determined in control (black bars) and *Cstb*^*-/-*^ microglia (grey bars). The asterisk (*) marks genes significantly altered in *Cstb*^*-/-*^ microglia compared to control microglia. Error bars represent standard deviation (SD).
(PDF)

S3 Fig RNA expression level of transcripts *Irf7*, *Stat1*, and *Irf9* in *Cstb*^*-/-*^ and control microglia measured by qPCR. Transcript levels of *Irf7* and *Stat1* are significantly reduced in *Cstb*^*-/-*^ microglia. RNA expression from *Cstb*^*-/-*^ and control microglia measured by qPCR and normalized to *Tbp* transcript level. The average mRNA expression (± standard deviation) of three or four independent samples is depicted (* ≤ 0.05, ** ≤ 0.01).
(PDF)

S1 Table Transcripts differentially expressed in *Cstb*^*-/-*^ microglia. In the microarray experiment, genes with an absolute fold change (FC) ≥ 1.3 and a p-value < 0.05 were considered differentially expressed and in the RNA-seq analysis a local false discovery rate (FDR) < 0.01 was used as cutoff. Genes are sorted by FC identified in the microarray analysis and asterisks (*) mark genes differentially expressed in the results of both methods.
(PDF)

S2 Table Sequencing reads obtained by RNA-seq for control and *Cstb*^*-/-*^ microglia samples. (PDF)

S3 Table Expected numbers of overlapping DEGs. The overlap is significantly higher than the expectation (odds ratio = 132.2, p < 2.2e-16 by Fisher’s Exact test for count data, expected numbers are given in parenthesis).
(PDF)

S4 Table Gene ontology terms enriched in differentially expressed genes in *Cstb*^*-/-*^ microglia identified by microarray. N = total number of genes, B = Genes associated with GO term, n = Genes in the target set, b = Genes in target set associated with GO term.
(PDF)

S5 Table Gene ontology terms enriched in differentially expressed genes in *Cstb*^*-/-*^ microglia identified by RNA-seq. N = total number of genes, B = Genes associated with GO term, n = Genes at the top of the list, b = Genes at the top associated with GO term.
(PDF)

S6 Table Pathways enriched in differentially expressed genes in *Cstb*^*-/-*^ microglia. (PDF)

S7 Table Regulators enriched in differentially expressed genes in *Cstb*^*-/-*^ microglia. (PDF)
