# Supplementary material for: Gene-Expression Profiling Suggests Impaired Signaling via the Interferon Pathway in Cstb-/- Microglia
Source: PLoS One. 2016 Jun 29;11(6):e0158195. doi: 10.1371/journal.pone.0158195 (PMC4927094; doi:10.1371/journal.pone.0158195)
Supplement: S1 Fig — The DEG expression level in (A) microarray and (B) RNA-seq. Microarray specific DEGs are depicted in violet, RNA-seq specific DEGs in light blue, and DEGs common to both methods in orange. (PDF) [file pone.0158195.s001.pdf]

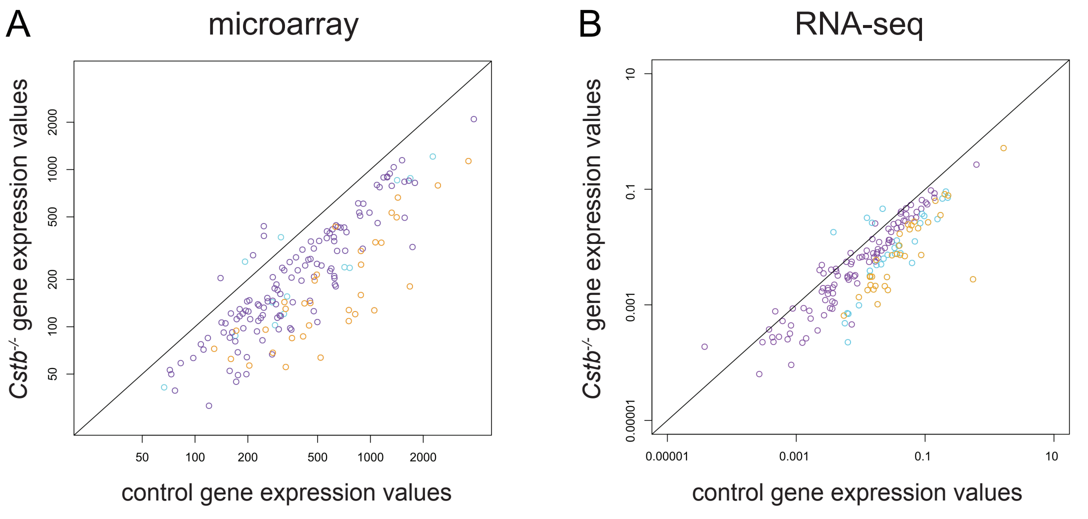
A
microarray
B
RNA-seq
Cstb-/- gene expression values
control gene expression values
50
100
200
500
1000
2000
0.00001
0.001
0.1
10
