# Supplementary material for: Gene-Expression Profiling Suggests Impaired Signaling via the Interferon Pathway in Cstb-/- Microglia
Source: PLoS One. 2016 Jun 29;11(6):e0158195. doi: 10.1371/journal.pone.0158195 (PMC4927094; doi:10.1371/journal.pone.0158195)
Supplement: S2 Fig — RNA-seq gene expression level of transcripts (A) reportedly enriched in microglia, (B) associated with pro-inflammatory, and (C) with anti-inflammatory activation of microglia determined in control (black bars) and Cstb-/- microglia (grey bars). The asterisk (*) marks genes significantly altered in Cstb-/- microglia compared to control microglia. Error bars represent standard deviation (SD). (PDF) [file pone.0158195.s002.pdf]

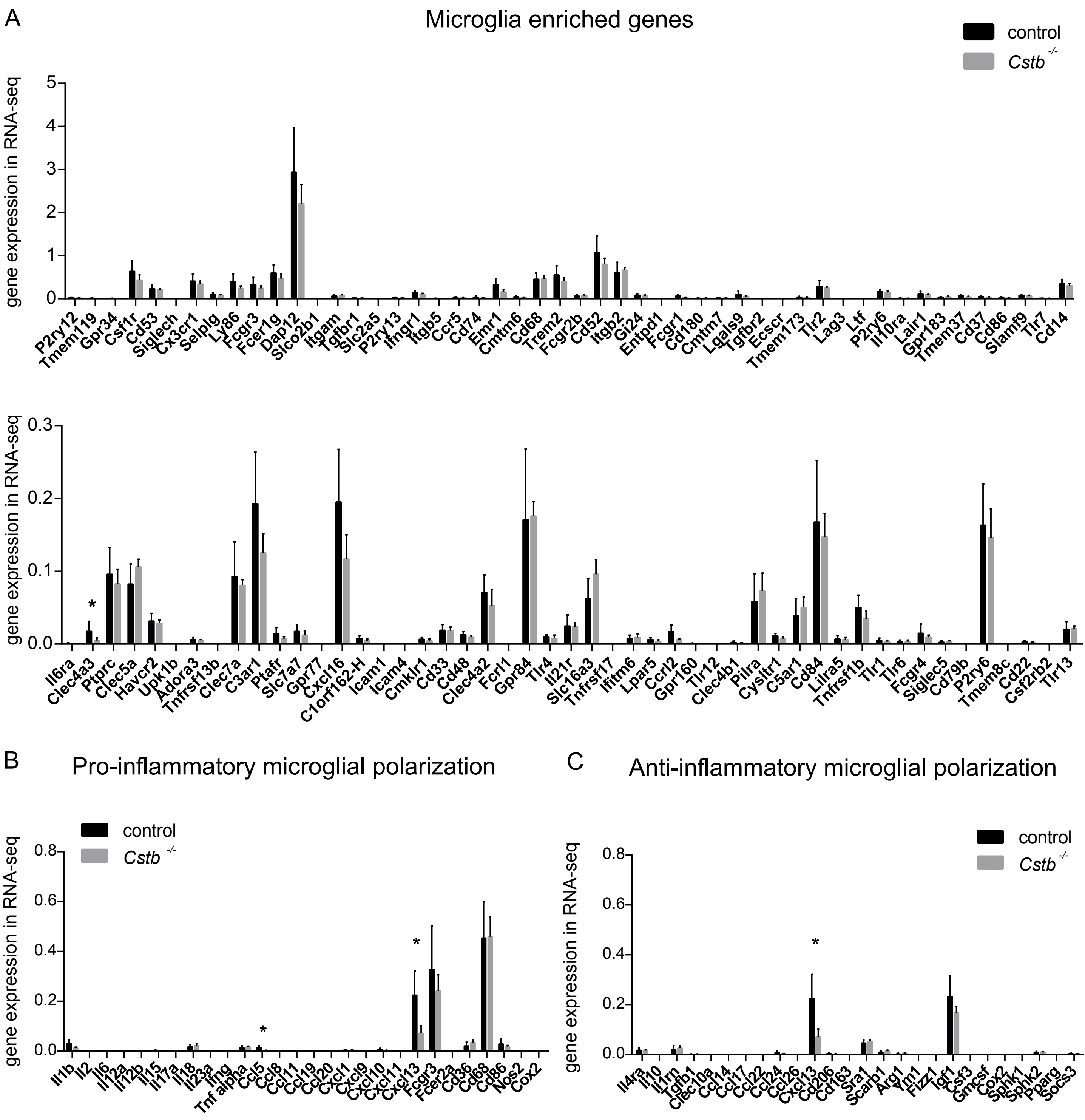
A
Microglia enriched genes
control
Cstb -/-
gene expression in RNA-seq
0 1 2 3 4 5
P2ry12 Tmem119 Gpr34 Csf1r Cd53 Siglech Cx3cr1 Selplg Ly86 Fcgr3 Fcer1g Dap12 Slco2b1 Itgam Tgfbr1 Slc2a5 P2ry13 Ifngr1 Itgb5 Ccr5 Cd74 Emr1 Cmtm6 Cd68 Trem2 Fcgr2b Cd52 Itgb2 Gi24 Entpd1 Fcgr1 Cd180 Cmtm7 Lgals9 Tgfbr2 Ecscr Tmem173 Tlr2 Lag3 Ltf P2ry6 Il10ra Lair1 Gpr183 Tmem37 Cd37 Cd86 Slamf9 Tlr7 Cd14
gene expression in RNA-seq
0.0 0.1 0.2 0.3
*
Il6ra Clec4a3 Ptprc Clec5a Havcr2 Upk1b Adora3 Tnfrsf13b Clec7a C3ar1 Ptafr Slc7a7 Gpr77 Cxcl16 C1orf162-H Icam1 Icam4 Cmklr1 Cd33 Cd48 Clec4a2 Fcrl1 Gpr84 Tlr4 Il21r Slc16a3 Tnfrsf17 Ifitm6 Lpar5 Ccrl2 Gpr160 Tlr12 Clec4b1 Pilra Cysltr1 C5ar1 Cd84 Lilra5 Tnfrsf1b Tlr1 Tlr6 Fcgr4 Siglec5 Cd79b P2ry6 Tmem8c Cd22 Csf2rb2 Tlr13
B
Pro-inflammatory microglial polarization
control
Cstb -/-
gene expression in RNA-seq
0.0 0.2 0.4 0.6 0.8
* *
Il1b Il2 Il6 Il12a Il12b Il15 Il17a Il18 Il23a Ifng Tnf alpha Ccl5 Ccl8 Ccl11 Ccl19 Ccl20 Cxcl1 Cxcl9 Cxcl10 Cxcl11 Cxcl13 Fcgr3 Fcer2a Cd36 Cd68 Cd86 Nos2 Cox2
C
Anti-inflammatory microglial polarization
control
Cstb -/-
gene expression in RNA-seq
0.0 0.2 0.4 0.6 0.8
*
Il4ra Il10 Il1rn Tgfb1 Clec10a Ccl14 Ccl17 Ccl22 Ccl24 Ccl26 Cxcl13 Cd206 Cd163 Sra1 Scarb1 Arg1 Ym1 Fizz1 Igf1 Csf3 Gmcsf Cox2 Sphk1 Sphk2 Pparg Socs3
