# Supplementary figures and images for: Gene-Expression Profiling Suggests Impaired Signaling via the Interferon Pathway in Cstb-/- Microglia
Source: PLoS One. 2016 Jun 29;11(6):e0158195. doi: 10.1371/journal.pone.0158195 (PMC4927094; doi:10.1371/journal.pone.0158195)

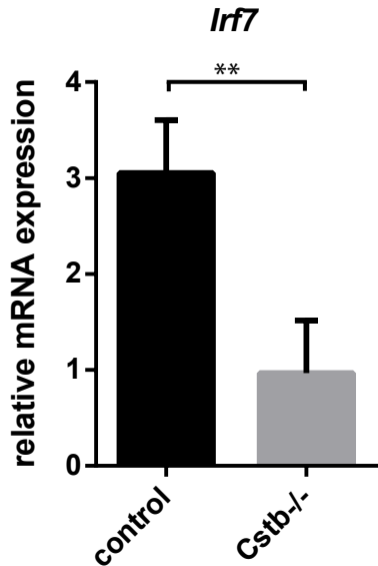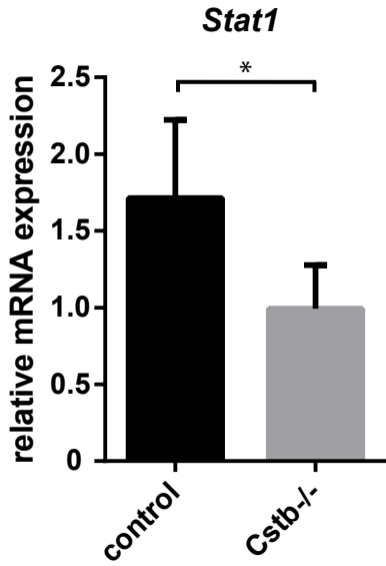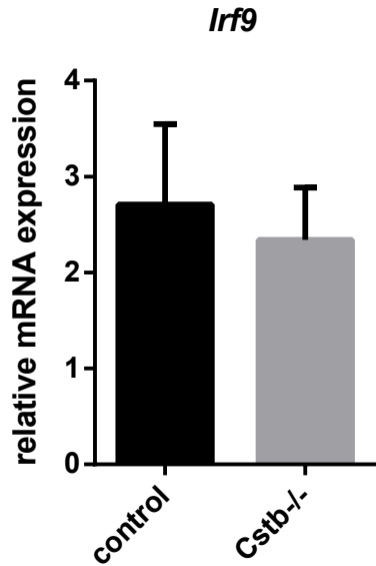

Supplement: S3 Fig — Transcript levels of Irf7 and Stat1 are significantly reduced in Cstb-/- microglia. RNA expression from Cstb-/- and control microglia measured by qPCR and normalized to Tbp transcript level. The average mRNA expression (± standard deviation) of three or four independent samples is depicted (* ≤ 0.05, ** ≤ 0.01). (PDF) [file pone.0158195.s003.pdf]
