# Supplementary material for: Gene-Expression Profiling Suggests Impaired Signaling via the Interferon Pathway in Cstb-/- Microglia
Source: PLoS One. 2016 Jun 29;11(6):e0158195. doi: 10.1371/journal.pone.0158195 (PMC4927094; doi:10.1371/journal.pone.0158195)
Supplement: S1 Table — In the microarray experiment, genes with an absolute fold change (FC) ≥ 1.3 and a p-value < 0.05 were considered differentially expressed and in the RNA-seq analysis a local false discovery rate (FDR) < 0.01 was used as cutoff. Genes are sorted by FC identified in the microarray analysis and asterisks (*) mark genes differentially expressed in the results of both methods. (PDF) [file pone.0158195.s004.pdf]

Supplementary Table 1: Transcripts differentially expressed in *Cstb* $^{-/-}$ microglia.

In the microarray experiment, genes with an absolute fold change (FC) ≥ 1.3 and a p-value < 0.05 were considered differentially expressed and in the RNA-seq analysis a local false discovery rate (FDR) < 0.01 was used as cutoff. Genes are sorted by FC identified in the microarray analysis and asterisks (*) mark genes differentially expressed in the results of both methods.

	microarray			RNA-seq	
**Gene Symbol**	**TC ID**	**FC**	**p-value**	**FC**	**Local FDR**
*Cstb**	6769080	-9.3	2.11E-06	-200.2	3.26E-16
*Ifit1**	6869334	-8.1	2.24E-02	-11.2	9.24E-14
*Ifit3**	6869327	-7.5	3.06E-02	-8.5	7.49E-13
*Slfn5**	6782979	-6.7	2.24E-02	-14.7	1.10E-16
*Oasl2**	6933625	-6.6	2.29E-02	-17.9	1.85E-09
*Rtp4**	6840129	-5.8	2.24E-02	-6.9	4.17E-11
*Mx1**	6848199	-5.5	3.26E-02	-6.9	2.14E-08
*Irf7**	6972192	-5.3	2.29E-02	-5.6	5.17E-10
Mnda|Ifi204|Mndal	6764246	-5.3	2.24E-02	n.d.	n.d.
Oas2	6941647	-4.5	2.24E-02	-3.8	n.s.
*Oasl1**	6933627	-4.5	3.41E-02	-7.1	4.19E-08
*Ifit2**	6869324	-4.1	4.25E-02	-8.0	9.91E-09
*Zbp1**	6893556	-4.0	3.26E-02	-8.6	9.33E-07
*Rsad2**	6799525	-3.9	2.81E-02	-6.4	2.88E-07
*Saa3**	6967059	-3.9	4.56E-02	-4.9	7.83E-06
Ms4a4c	6868091	-3.9	3.93E-02	-3.2	n.s.
Ifi205	6764243	-3.8	3.46E-02	n.d.	n.d.
Mx2	6843550	-3.7	2.81E-02	n.d.	n.d.
D14Ertd668e|Phf11	6828125	-3.7	2.50E-02	n.d.	n.d.
Pyhin1	6764231	-3.6	4.24E-02	-2.5	n.s.
Clec4a1	6949722	-3.6	n.s.	-4.1	7.62E-03
Emr4	6851186	-3.5	2.81E-02	-4.2	n.s.
Gbp6	6901950	-3.5	4.24E-02	-4.2	n.s.
Ifi202b	6764289	-3.4	2.24E-02	-1.9	n.s.
Phf11	6825096	-3.4	3.16E-02	-3.8	n.s.
Dhx58	6791437	-3.4	2.30E-02	n.d.	n.d.
*Gbp2**	6901957	-3.4	4.24E-02	-4.9	2.21E-04
*Ddx58**	6920954	-3.3	2.24E-02	-3.7	4.48E-06
*Ifi44**	6910592	-3.2	1.55E-03	-4.8	3.14E-09
Clec4a3	6949727	-3.2	n.s.	-3.6	9.55E-05
Rnf213	6785384	-3.2	2.24E-02	-1.6	n.s.
*Ifi27l2a**	6803210	-3.1	2.56E-02	-3.2	7.83E-06
*Parp14**	6845375	-3.1	2.24E-02	-4.2	4.19E-08
*Lgals3bp**	6792657	-3.1	2.24E-02	-3.7	8.17E-16
Lcn2	6885873	-3.0	2.45E-02	-15.9	n.s.
Ifih1	6887196	-3.0	2.45E-02	-2.5	n.s.
*Sp100**	6751219	-3.0	2.81E-02	-2.6	1.27E-04
Cmpk2	6793961	-3.0	3.93E-02	-5.6	n.s.
Cxcl10	6939990	-3.0	3.26E-02	-2.4	n.s.
Oas1a|Oas1g	6941657	-2.9	2.24E-02	n.d.	n.d.
Dtx3l	6845377	-2.9	2.71E-02	-2.4	n.s.

*Irgm1**	6787896	-2.9	2.50E-02	-2.9	5.75E-03
Samd9l	6951281	-2.9	3.49E-02	-6.0	n.s.
Igtp|Gm12250|Irgm2	6781248	-2.9	3.08E-02	n.d.	n.d.
*Stat1**	6749376	-2.8	2.29E-02	-4.2	3.06E-13
Aoah	6805180	-2.7	4.56E-02	-2.3	n.s.
Trim34	6963137	-2.6	2.29E-02	n.d.	n.d.
*Ccl5**	6790288	-2.6	2.46E-02	-4.6	2.69E-04
Isg20	6961896	-2.6	n.s.	-9.2	2.88E-03
BC006779	6894274	-2.6	2.29E-02	-2.3	n.s.
Epsti1	6820472	-2.6	2.56E-02	-2.9	n.s.
*Slfn8**	6790244	-2.5	2.24E-02	-4.6	3.85E-03
Stat2	6771641	-2.5	2.29E-02	-2.3	n.s.
Il1b	6890838	-2.5	2.29E-02	-2.5	n.s.
Clec2d	6950125	-2.5	3.60E-02	-2.5	n.s.
*Oas1b**	6933997	-2.5	4.25E-02	-8.4	1.99E-03
*Tor3a**	6763273	-2.5	2.24E-02	-2.5	1.32E-03
*Parp9**	6840734	-2.5	2.29E-02	-2.4	9.12E-03
Apoc1	6973586	-2.4	n.s.	-5.1	4.46E-10
Parp12	6952926	-2.4	2.24E-02	n.d.	n.d.
P2ry14	6905408	-2.4	3.67E-02	-1.6	n.s.
C3	6856290	-2.4	4.82E-02	-3.6	n.s.
Eif2ak2	6857435	-2.3	2.24E-02	-2.4	n.s.
*Ube2l6**	6878709	-2.3	3.23E-02	-2.2	4.11E-03
*Bst2**	6983265	-2.2	3.74E-02	-2.9	8.36E-07
Trim30	6970065	-2.2	2.55E-02	n.d.	n.d.
*Slfn2**	6782988	-2.2	2.24E-02	-2.3	8.50E-04
Gvin1|Gm8979	6970164	-2.1	2.81E-02	n.d.	n.d.
H2-T10|H2-T22|H2-T23|H2-T24	6855152	-2.1	2.29E-02	n.d.	n.d.
Cyp27a1	6750566	-2.1	n.s.	-3.6	2.54E-03
Slfn10-ps	6790245	-2.1	2.29E-02	-1.1	n.s.
Ccrl2	6998919	-2.1	2.74E-02	-3.0	n.s.
Tap1	6850019	-2.1	3.15E-02	-2.0	n.s.
Uba7|Cdh29|Cdhr4	6992280	-2.1	3.23E-02	n.d.	n.d.
Slfn9	6790239	-2.0	2.38E-02	-2.9	n.s.
Sepp1	6828326	-2.0	n.s.	-2.3	1.30E-04
Pml	6995964	-2.0	2.84E-02	1.3	n.s.
Ms4a7	6871545	-2.0	4.68E-02	-1.9	n.s.
Lgals9	6790012	-2.0	2.81E-02	-2.0	n.s.
Cxcl13	6932571	-1.9	n.s.	-3.1	2.80E-04
Casp4	6986649	-1.9	3.06E-02	n.d.	n.d.
Parp10	6836806	-1.9	2.50E-02	-2.1	n.s.
Trim25	6783439	-1.9	3.06E-02	-1.7	n.s.
Irf1	6780996	-1.9	4.21E-02	-2.5	n.s.
Ly6e	6831538	-1.9	3.60E-02	-2.3	n.s.
Tmem140	6945339	-1.9	4.56E-02	-2.4	n.s.
Fcgr1	6907262	-1.9	3.93E-02	-2.0	n.s.
Zufsp	6773158	-1.9	4.24E-02	-1.6	n.s.
Abca1	6921670	-1.9	2.71E-02	-9.1	n.s.
Cd83	6806640	-1.8	n.s.	-3.5	1.41E-04
Timd4	6780572	-1.8	3.49E-02	-2.1	n.s.

Abcg1	6849766	-1.8	4.56E-02	-2.4	n.s.
*Trex1**	6998741	-1.8	2.24E-02	-2.4	1.65E-04
Map3k8	6863048	-1.8	4.65E-02	n.d.	n.d.
Ikbke	6762151	-1.8	4.56E-02	-1.4	n.s.
Gpr18	6827943	-1.8	n.s.	-8.7	7.50E-05
Gdap10	6794294	-1.8	2.81E-02	n.d.	n.d.
*Isg15**	6927363	-1.8	3.93E-02	-11.9	1.67E-19
9930111J21Rik2	6787925	-1.7	4.68E-02	n.d.	n.d.
Ifi35	6784270	-1.7	2.55E-02	-1.9	n.s.
Adar	6899262	-1.7	2.24E-02	-1.7	n.s.
Fbxw17	6806991	-1.7	4.53E-02	-2.1	n.s.
Trim14	6921379	-1.7	4.83E-02	-1.4	n.s.
Slc39a4	6836839	-1.7	3.93E-02	-2.0	n.s.
Tapbp	6849990	-1.7	3.63E-02	-1.8	n.s.
Psmb9	6855013	-1.7	3.60E-02	-1.8	n.s.
P2rx4	6934164	-1.7	n.s.	-2.4	2.96E-03
Mov10	6907869	-1.7	3.60E-02	-1.5	n.s.
Arid5b	6774719	-1.7	2.38E-02	-1.9	n.s.
Nampt	6794293	-1.7	2.29E-02	-2.0	n.s.
Phyhd1|Lrrc8a	6876052	-1.6	4.81E-02	n.d.	n.d.
Car13	6895838	-1.6	4.53E-02	-2.2	n.s.
H2-gs10	6850155	-1.6	3.88E-02	-1.0	n.s.
Trafd1	6941672	-1.6	3.06E-02	-1.6	n.s.
H2-T22|H2-T10|H2-T9	7005505	-1.6	3.67E-02	n.d.	n.d.
Irf9	6819258	-1.6	3.46E-02	-1.6	n.s.
H2-T10	6855155	-1.6	3.60E-02	5.0	n.s.
Nod1	6953800	-1.6	4.56E-02	-1.5	n.s.
Tmcc3	6769928	-1.6	4.90E-02	-1.0	n.s.
Sult1a1	6971280	-1.5	n.s.	-28.1	2.18E-03
1600014C10Rik	6959968	-1.5	4.56E-02	-2.2	n.s.
Tnfaip3	6772417	-1.5	1.96E-02	-2.9	n.s.
Blnk	6873111	-1.5	4.81E-02	-1.6	n.s.
Prdm1	6773655	-1.5	2.29E-02	-2.0	n.s.
Optn	6884446	-1.5	2.55E-02	-1.8	n.s.
Rxrg	6754900	-1.5	3.26E-02	-1.5	n.s.
Sass6	6900713	-1.5	3.05E-02	1.2	n.s.
Stard3	6783998	-1.5	3.60E-02	-1.3	n.s.
Rnf114	6883320	-1.5	4.56E-02	-1.8	n.s.
Slc29a3	6774309	-1.5	3.88E-02	-1.3	n.s.
Trim12|9230105E10Rik|Trim5	6970053	-1.5	3.88E-02	n.d.	n.d.
*Tor1aip1**	6763240	-1.5	3.05E-02	-2.8	1.04E-03
Ptger4	6833640	-1.5	2.50E-02	-2.0	n.s.
Chmp4b	6882476	-1.5	3.92E-02	-5.4	n.s.
Ankle2	6933409	-1.4	3.49E-02	n.d.	n.d.
Ftsjd2	6849665	-1.4	2.29E-02	-1.4	n.s.
Cds1	6932930	-1.4	4.98E-02	n.d.	n.d.
Tmem219	6971307	-1.4	3.60E-02	-1.4	n.s.
Cd22	6966322	-1.4	4.49E-02	-1.7	n.s.
Tnfrsf26	6972411	-1.4	4.56E-02	1.6	n.s.
Cnot6l	6940146	-1.4	3.45E-02	1.1	n.s.

Pvrl4	6755175	-1.4	4.48E-02	-4.1	n.s.
Ogfr	6884183	-1.4	2.81E-02	-1.1	n.s.
Gab2	6962745	-1.4	4.47E-02	-1.3	n.s.
Sdc3	6917389	-1.4	4.56E-02	-1.9	n.s.
Ly9	6764089	-1.4	4.56E-02	-1.1	n.s.
Cd86	6845435	-1.4	4.56E-02	-1.6	n.s.
Cd47	6841410	-1.4	3.60E-02	-1.4	n.s.
Acp2	6879034	-1.4	3.88E-02	-1.4	n.s.
Eng	6876212	-1.4	3.92E-02	-1.3	n.s.
Rnf31	6819257	-1.4	3.60E-02	-1.3	n.s.
Hpse	6940363	-1.4	4.53E-02	n.d.	n.d.
Ascc3	6767760	-1.3	4.53E-02	-1.9	n.s.
Dck	6932234	-1.3	3.88E-02	-1.1	n.s.
Sema4d	6813187	-1.3	4.48E-02	-1.0	n.s.
Rab20	6980378	-1.3	2.24E-02	-1.7	n.s.
Nub1	6929312	-1.3	3.88E-02	-1.1	n.s.
Tcirg1	6870956	-1.3	3.88E-02	-1.1	n.s.
Ttc39b	6922895	-1.3	3.60E-02	1.0	n.s.
Mobkl2c	6916510	-1.3	4.65E-02	-2.1	n.s.
Fndc3a	6825853	-1.3	2.56E-02	-1.2	n.s.
Slc15a3	6868058	-1.3	3.93E-02	-1.3	n.s.
Bysl	6855772	1.3	3.21E-02	1.5	n.s.
Ccl2	6782915	1.4	n.s.	2.6	7.28E-05
Ccr1	6999688	1.4	n.s.	2.1	3.24E-03
Slc39a14	6825688	1.5	2.50E-02	1.7	n.s.
Pcdh7	6931001	1.5	3.87E-02	2.0	n.s.
Lpcat4	6880033	1.8	3.49E-02	n.d.	n.d.
Gm14446	n.d.	n.d.	n.d.	-11.8	4.71E-06
Ifit3	n.d.	n.d.	n.d.	-11.8	2.89E-24
Gm12250	n.d.	n.d.	n.d.	-9.6	1.16E-04
H2-T24	n.d.	n.d.	n.d.	-5.5	6.68E-13
Usp18	n.d.	n.d.	n.d.	-5.3	7.58E-07
Gm4902	n.d.	n.d.	n.d.	-4.5	1.85E-09
Cd300lh	n.d.	n.d.	n.d.	-4.4	7.58E-03
Xaf1	n.d.	n.d.	n.d.	-3.7	3.75E-05
Oas1a	n.d.	n.d.	n.d.	-3.6	4.78E-04
Herc6	n.d.	n.d.	n.d.	-3.6	2.68E-05
H2-T23	n.d.	n.d.	n.d.	-3.2	2.18E-07
Lst1	n.d.	n.d.	n.d.	-2.8	2.29E-04
AI607873	n.d.	n.d.	n.d.	-2.8	2.59E-04
Tmem176a	n.d.	n.d.	n.d.	-2.7	2.06E-04
Ptpn7	n.d.	n.d.	n.d.	1.8	7.00E-03
Serpinb10-ps	n.d.	n.d.	n.d.	4.8	1.01E-04

TC ID = Transcript cluster Id, n.d. = not determined, n.s. = not significant
