# Supplementary material for: Gene-Expression Profiling Suggests Impaired Signaling via the Interferon Pathway in Cstb-/- Microglia
Source: PLoS One. 2016 Jun 29;11(6):e0158195. doi: 10.1371/journal.pone.0158195 (PMC4927094; doi:10.1371/journal.pone.0158195)
Supplement: S2 Table — (PDF) [file pone.0158195.s005.pdf]

Supplementary table 2: Sequencing reads obtained by RNA-seq for control and *Cstb*$^{-/-}$ microglia samples.

sample	repeat	qualified reads	total reads	redundancy	mapped reads	mapped rate	spike-in reads	mapped / spike-in
control 1	1	2984694	679195	4.4	606601	0.89	564	1076
control 1	2	2317592	517534	4.5	457869	0.88	552	829
control 1	3	2527847	559275	4.5	491439	0.88	625	786
control 1	4	1714206	396263	4.3	353819	0.89	432	819
control 2	1	2500907	553827	4.5	492702	0.89	1299	379
control 2	2	2252758	506434	4.4	443755	0.88	1279	347
control 2	3	1899071	452731	4.2	396990	0.88	1034	384
control 2	4	1779932	397579	4.5	353190	0.89	834	423
control 3	1	2553812	566561	4.5	498987	0.88	1279	390
control 3	2	3107175	643850	4.8	563026	0.87	877	642
control 3	3	2781654	591424	4.7	518123	0.88	717	723
control 3	4	2205093	459893	4.8	406846	0.88	660	616
control 4	1	2431170	597674	4.1	526259	0.88	1726	305
control 4	2	2574585	565357	4.6	501508	0.89	1070	469
control 4	3	2656682	581931	4.6	516519	0.89	919	562
control 4	4	2467864	542767	4.5	484953	0.89	715	678
Cstb-/- 1	1	2866067	605222	4.7	537135	0.89	1175	457
Cstb-/- 1	2	2425846	523996	4.6	462365	0.88	1041	444
Cstb-/- 1	3	1726517	373496	4.6	329673	0.88	647	510
Cstb-/- 1	4	3084003	649858	4.7	575629	0.89	727	792
Cstb-/- 2	1	3944327	794888	5.0	699396	0.88	1331	525
Cstb-/- 2	2	1887789	432366	4.4	384937	0.89	895	430
Cstb-/- 2	3	2257267	528927	4.3	468471	0.89	752	623
Cstb-/- 2	4	1995105	469485	4.2	416751	0.89	673	619
Cstb-/- 3	1	2352112	531547	4.4	472982	0.89	1099	430
Cstb-/- 3	2	1959027	441155	4.4	392178	0.89	910	431
Cstb-/- 3	3	2728797	588194	4.6	521859	0.89	943	553
Cstb-/- 3	4	2810710	595598	4.7	527292	0.89	702	751
Cstb-/- 4	1	1979259	454647	4.4	405341	0.89	493	822
Cstb-/- 4	2	1763589	418578	4.2	372098	0.89	694	536
Cstb-/- 4	3	2169921	504735	4.3	448723	0.89	574	782
Cstb-/- 4	4	2258327	507985	4.4	449300	0.88	522	861

sample	repeat	spike-in 5-end reads	spike-in 5-end rate	coding reads	coding 5-end reads	coding 5-end rate	force approval
control 1	1	538	0.95	539076	431398	0.80	FALSE
control 1	2	523	0.95	401998	321253	0.80	FALSE
control 1	3	602	0.96	429162	346765	0.81	FALSE
control 1	4	406	0.94	314010	253953	0.81	FALSE
control 2	1	1219	0.94	436281	351597	0.81	FALSE
control 2	2	1213	0.95	382418	306825	0.80	FALSE
control 2	3	982	0.95	339670	273879	0.81	FALSE
control 2	4	797	0.96	310564	244472	0.79	FALSE
control 3	1	1206	0.94	432540	347957	0.80	FALSE
control 3	2	828	0.94	486974	391656	0.80	FALSE
control 3	3	675	0.94	444698	356105	0.80	FALSE
control 3	4	620	0.94	357503	290188	0.81	FALSE
control 4	1	1653	0.96	452748	361678	0.80	FALSE
control 4	2	1030	0.96	440975	357697	0.81	FALSE
control 4	3	872	0.95	452378	364774	0.81	FALSE
control 4	4	670	0.94	429548	347108	0.81	FALSE
Cstb-/- 1	1	1129	0.96	478488	394049	0.82	FALSE
Cstb-/- 1	2	986	0.95	408077	336150	0.82	FALSE
Cstb-/- 1	3	616	0.95	291861	240904	0.83	FALSE
Cstb-/- 1	4	684	0.94	513870	423753	0.82	FALSE
Cstb-/- 2	1	1248	0.94	599752	475204	0.79	FALSE
Cstb-/- 2	2	851	0.95	334837	267840	0.80	FALSE
Cstb-/- 2	3	721	0.96	407345	328726	0.81	FALSE
Cstb-/- 2	4	636	0.95	361631	290309	0.80	FALSE
Cstb-/- 3	1	1036	0.94	424043	348208	0.82	FALSE
Cstb-/- 3	2	850	0.93	351672	290626	0.83	FALSE
Cstb-/- 3	3	890	0.94	468891	389908	0.83	FALSE
Cstb-/- 3	4	658	0.94	471729	387096	0.82	FALSE
Cstb-/- 4	1	465	0.94	356305	285920	0.80	FALSE
Cstb-/- 4	2	648	0.93	326924	265279	0.81	FALSE
Cstb-/- 4	3	544	0.95	395855	320891	0.81	FALSE
Cstb-/- 4	4	483	0.93	392434	311760	0.79	FALSE

sample	repeat	spike-in reads outlier	mapped / spike-in outlier	spike-in 5-end rate outlier	coding 5-end rate outlier
control 1	1	FALSE	FALSE	FALSE	FALSE
control 1	2	FALSE	FALSE	FALSE	FALSE
control 1	3	FALSE	FALSE	FALSE	FALSE
control 1	4	FALSE	FALSE	FALSE	FALSE
control 2	1	FALSE	FALSE	FALSE	FALSE
control 2	2	FALSE	FALSE	FALSE	FALSE
control 2	3	FALSE	FALSE	FALSE	FALSE
control 2	4	FALSE	FALSE	FALSE	FALSE
control 3	1	FALSE	FALSE	FALSE	FALSE
control 3	2	FALSE	FALSE	FALSE	FALSE
control 3	3	FALSE	FALSE	FALSE	FALSE
control 3	4	FALSE	FALSE	FALSE	FALSE
control 4	1	FALSE	FALSE	FALSE	FALSE
control 4	2	FALSE	FALSE	FALSE	FALSE
control 4	3	FALSE	FALSE	FALSE	FALSE
control 4	4	FALSE	FALSE	FALSE	FALSE
Cstb-/- 1	1	FALSE	FALSE	FALSE	FALSE
Cstb-/- 1	2	FALSE	FALSE	FALSE	FALSE
Cstb-/- 1	3	FALSE	FALSE	FALSE	FALSE
Cstb-/- 1	4	FALSE	FALSE	FALSE	FALSE
Cstb-/- 2	1	FALSE	FALSE	FALSE	FALSE
Cstb-/- 2	2	FALSE	FALSE	FALSE	FALSE
Cstb-/- 2	3	FALSE	FALSE	FALSE	FALSE
Cstb-/- 2	4	FALSE	FALSE	FALSE	FALSE
Cstb-/- 3	1	FALSE	FALSE	FALSE	FALSE
Cstb-/- 3	2	FALSE	FALSE	FALSE	FALSE
Cstb-/- 3	3	FALSE	FALSE	FALSE	FALSE
Cstb-/- 3	4	FALSE	FALSE	FALSE	FALSE
Cstb-/- 4	1	FALSE	FALSE	FALSE	FALSE
Cstb-/- 4	2	FALSE	FALSE	FALSE	FALSE
Cstb-/- 4	3	FALSE	FALSE	FALSE	FALSE
Cstb-/- 4	4	FALSE	FALSE	FALSE	FALSE
