# Supplementary material for: Gene-Expression Profiling Suggests Impaired Signaling via the Interferon Pathway in Cstb-/- Microglia
Source: PLoS One. 2016 Jun 29;11(6):e0158195. doi: 10.1371/journal.pone.0158195 (PMC4927094; doi:10.1371/journal.pone.0158195)
Supplement: S3 Table — The overlap is significantly higher than the expectation (odds ratio = 132.2, p < 2.2e-16 by Fisher’s Exact test for count data, expected numbers are given in parenthesis). (PDF) [file pone.0158195.s006.pdf]

Supplementary table 3: Expected number of overlapping DEGs.

	Microarray DEG	Microarray non-DEG or not detected	Total
**RNA-seq DEG**	33 (1)	122 (154)	155
**RNA-seq non-DEG or not detected**	29 (61)	14236 (14204)	14265
	62	14358	14420
