# Supplementary material for: Gene-Expression Profiling Suggests Impaired Signaling via the Interferon Pathway in Cstb-/- Microglia
Source: PLoS One. 2016 Jun 29;11(6):e0158195. doi: 10.1371/journal.pone.0158195 (PMC4927094; doi:10.1371/journal.pone.0158195)
Supplement: S4 Table — N = total number of genes, B = Genes associated with GO term, n = Genes in the target set, b = Genes in target set associated with GO term. (PDF) [file pone.0158195.s007.pdf]

Supplementary table 4: Gene ontology terms enriched in differentially expressed genes in *Cstb* $^{-/-}$ microglia identified by microarray.

N = total number of genes, B = Genes associated with GO term, n = Genes in the target set, b = Genes in target set associated with GO term

Biological processes

**GO Term**	**Description**	**p-value**	**FDR q-value**	**Enrichment**	**N**	**B**	**n**	**b**
GO:0009615	response to virus	3.49E-27	4.66E-23	20.84	18026	147	153	26
GO:0043207	response to external biotic stimulus	1.05E-24	7.01E-21	8.43	18026	531	153	38
GO:0051707	response to other organism	3.32E-24	1.48E-20	10.31	18026	377	153	33
GO:0009607	response to biotic stimulus	4.80E-24	1.60E-20	8.08	18026	554	153	38
GO:0051607	defense response to virus	9.90E-24	2.65E-20	22.15	18026	117	153	22
GO:0006952	defense response	2.87E-21	6.40E-18	6.28	18026	751	153	40
GO:0002252	immune effector process	4.52E-21	8.64E-18	10.75	18026	307	153	28
GO:0051704	multi-organism process	1.93E-20	3.23E-17	6.89	18026	616	153	36
GO:0098542	defense response to other organism	1.05E-19	1.56E-16	10.16	18026	313	153	27
GO:0045087	innate immune response	4.75E-19	6.35E-16	10.21	18026	300	153	26
GO:0002376	immune system process	5.92E-19	7.20E-16	4.81	18026	1077	153	44
GO:0009605	response to external stimulus	1.44E-17	1.60E-14	5.09	18026	903	153	39
GO:0006955	immune response	4.52E-17	4.65E-14	6.58	18026	555	153	31
GO:0048525	negative regulation of viral process	5.04E-17	4.82E-14	23.88	18026	74	153	15
GO:0043901	negative regulation of multi-organism process	5.67E-17	5.05E-14	16.07	18026	132	153	18
GO:0035456	response to interferon-beta	3.47E-16	2.90E-13	44.69	18026	29	153	11
GO:0050792	regulation of viral process	5.21E-16	4.10E-13	15.9	18026	126	153	17
GO:0035458	cellular response to interferon-beta	4.22E-15	3.14E-12	47.13	18026	25	153	10
GO:0043903	regulation of symbiosis, encompassing mutualism through parasitism	1.78E-14	1.26E-11	12.92	18026	155	153	17
GO:1903901	negative regulation of viral life cycle	2.22E-14	1.48E-11	21.88	18026	70	153	13
GO:1903900	regulation of viral life cycle	8.27E-14	5.27E-11	14.85	18026	119	153	15
GO:0045071	negative regulation of viral genome replication	1.59E-13	9.65E-11	34.65	18026	34	153	10
GO:0043900	regulation of multi-organism process	2.37E-13	1.38E-10	7.41	18026	350	153	22
GO:0034097	response to cytokine	3.46E-13	1.93E-10	9.03	18026	248	153	19
GO:0031347	regulation of defense response	1.05E-12	5.60E-10	6.13	18026	461	153	24
GO:0006950	response to stress	1.14E-12	5.87E-10	2.9	18026	2030	153	50
GO:0045088	regulation of innate immune response	1.74E-11	8.62E-09	11.62	18026	142	153	14
GO:0045069	regulation of viral genome replication	3.68E-11	1.76E-08	21.04	18026	56	153	10

GO:0002682	regulation of immune system process	2.50E-10	1.15E-07	3.66	18026	997	153	31
GO:0071345	cellular response to cytokine stimulus	2.63E-10	1.17E-07	10.71	18026	143	153	13
GO:0050776	regulation of immune response	3.65E-09	1.58E-06	5.27	18026	425	153	19
GO:0001817	regulation of cytokine production	8.00E-09	3.35E-06	5.02	18026	446	153	19
GO:0045824	negative regulation of innate immune response	9.82E-09	3.98E-06	24.99	18026	33	153	7
GO:0002684	positive regulation of immune system process	3.49E-08	1.37E-05	4.14	18026	597	153	21
GO:0080134	regulation of response to stress	4.15E-08	1.59E-05	3.19	18026	1033	153	28
GO:0031349	positive regulation of defense response	9.01E-08	3.35E-05	7.36	18026	192	153	12
GO:0035455	response to interferon-alpha	1.67E-07	6.04E-05	36.82	18026	16	153	5
GO:0001819	positive regulation of cytokine production	2.18E-07	7.66E-05	5.61	18026	294	153	14
GO:0050896	response to stimulus	4.92E-07	1.69E-04	1.74	18026	4470	153	66
GO:0002474	antigen processing and presentation of peptide antigen via MHC class I	7.48E-07	2.50E-04	18.6	18026	38	153	6
GO:0010033	response to organic substance	7.88E-07	2.57E-04	2.58	18026	1413	153	31
GO:0032496	response to lipopolysaccharide	7.93E-07	2.52E-04	6.71	18026	193	153	11
GO:0031348	negative regulation of defense response	1.28E-06	3.97E-04	8.42	18026	126	153	9
GO:0002697	regulation of immune effector process	1.43E-06	4.35E-04	4.79	18026	344	153	14
GO:0002237	response to molecule of bacterial origin	1.58E-06	4.69E-04	6.26	18026	207	153	11
GO:0071310	cellular response to organic substance	2.97E-06	8.62E-04	3.16	18026	783	153	21
GO:0071222	cellular response to lipopolysaccharide	3.12E-06	8.87E-04	8.98	18026	105	153	8
GO:0050777	negative regulation of immune response	3.35E-06	9.33E-04	8.89	18026	106	153	8
GO:0032481	positive regulation of type I interferon production	3.46E-06	9.45E-04	21.04	18026	28	153	5
GO:0032020	ISG15-protein conjugation	5.92E-06	1.58E-03	70.69	18026	5	153	3
GO:0071219	cellular response to molecule of bacterial origin	6.16E-06	1.61E-03	8.2	18026	115	153	8
GO:0048002	antigen processing and presentation of peptide antigen	6.25E-06	1.61E-03	13.09	18026	54	153	6
GO:0019882	antigen processing and presentation	7.84E-06	1.98E-03	9.7	18026	85	153	7
GO:0051716	cellular response to stimulus	1.60E-05	3.95E-03	2.16	18026	1800	153	33
GO:0071216	cellular response to biotic stimulus	1.70E-05	4.13E-03	7.14	18026	132	153	8
GO:0032479	regulation of type I interferon production	1.88E-05	4.49E-03	15.1	18026	39	153	5
GO:0034121	regulation of toll-like receptor signaling pathway	1.88E-05	4.42E-03	15.1	18026	39	153	5
GO:0002683	negative regulation of immune system process	2.71E-05	6.24E-03	4.26	18026	332	153	12
GO:0042221	response to chemical	2.92E-05	6.62E-03	2.13	18026	1770	153	32
GO:0070887	cellular response to chemical stimulus	3.06E-05	6.82E-03	2.63	18026	986	153	22
GO:0032652	regulation of interleukin-1 production	3.06E-05	6.71E-03	13.7	18026	43	153	5

GO:0022610	biological adhesion	3.47E-05	7.49E-03	2.87	18026	780	153	19
GO:0032728	positive regulation of interferon-beta production	4.65E-05	9.86E-03	19.64	18026	24	153	4
GO:0050778	positive regulation of immune response	6.21E-05	1.30E-02	4.24	18026	306	153	11
GO:0098586	cellular response to virus	6.89E-05	1.42E-02	35.35	18026	10	153	3
GO:0002675	positive regulation of acute inflammatory response	8.72E-05	1.77E-02	16.83	18026	28	153	4
GO:0060338	regulation of type I interferon-mediated signaling pathway	9.41E-05	1.88E-02	32.13	18026	11	153	3
GO:0050729	positive regulation of inflammatory response	9.72E-05	1.91E-02	8.13	18026	87	153	6
GO:1903036	positive regulation of response to wounding	1.16E-04	2.26E-02	6.39	18026	129	153	7
GO:0032101	regulation of response to external stimulus	1.17E-04	2.24E-02	2.81	18026	712	153	17
GO:0050727	regulation of inflammatory response	1.32E-04	2.48E-02	4.71	18026	225	153	9
GO:0002698	negative regulation of immune effector process	1.33E-04	2.46E-02	7.68	18026	92	153	6
GO:0044764	multi-organism cellular process	1.33E-04	2.43E-02	7.68	18026	92	153	6
GO:0048583	regulation of response to stimulus	1.57E-04	2.84E-02	1.76	18026	2750	153	41
GO:0051092	positive regulation of NF-kappaB transcription factor activity	1.58E-04	2.82E-02	7.44	18026	95	153	6
GO:0032648	regulation of interferon-beta production	1.69E-04	2.97E-02	14.28	18026	33	153	4
GO:0002716	negative regulation of natural killer cell mediated immunity	2.04E-04	3.54E-02	25.25	18026	14	153	3
GO:0045953	negative regulation of natural killer cell mediated cytotoxicity	2.04E-04	3.49E-02	25.25	18026	14	153	3
GO:0032727	positive regulation of interferon-alpha production	2.04E-04	3.45E-02	25.25	18026	14	153	3
GO:0042089	cytokine biosynthetic process	2.04E-04	3.41E-02	25.25	18026	14	153	3
GO:0034340	response to type I interferon	2.14E-04	3.53E-02	78.54	18026	3	153	2
GO:0034124	regulation of MyD88-dependent toll-like receptor signaling pathway	2.14E-04	3.48E-02	78.54	18026	3	153	2
GO:0060333	interferon-gamma-mediated signaling pathway	2.14E-04	3.44E-02	78.54	18026	3	153	2
GO:0032651	regulation of interleukin-1 beta production	2.38E-04	3.79E-02	13.09	18026	36	153	4
GO:0002703	regulation of leukocyte mediated immunity	2.84E-04	4.46E-02	5.54	18026	149	153	7
GO:0007155	cell adhesion	3.05E-04	4.74E-02	2.59	18026	772	153	17
GO:0032647	regulation of interferon-alpha production	3.10E-04	4.76E-02	22.09	18026	16	153	3
GO:0032692	negative regulation of interleukin-1 production	3.10E-04	4.70E-02	22.09	18026	16	153	3
GO:0060759	regulation of response to cytokine stimulus	3.20E-04	4.81E-02	8.42	18026	70	153	5
GO:0002706	regulation of lymphocyte mediated immunity	3.34E-04	4.97E-02	6.49	18026	109	153	6
GO:0034341	response to interferon-gamma	3.96E-04	5.82E-02	11.49	18026	41	153	4
GO:1901342	regulation of vasculature development	4.20E-04	6.11E-02	4.51	18026	209	153	8
GO:0032367	intracellular cholesterol transport	4.25E-04	6.11E-02	58.91	18026	4	153	2
GO:1900225	regulation of NLRP3 inflammasome complex assembly	4.25E-04	6.04E-02	58.91	18026	4	153	2

GO:0039528	cytoplasmic pattern recognition receptor signaling pathway in response to virus	4.25E-04	5.98E-02	58.91	18026	4	153	2
GO:0001911	negative regulation of leukocyte mediated cytotoxicity	4.46E-04	6.21E-02	19.64	18026	18	153	3
GO:0042107	cytokine metabolic process	4.46E-04	6.14E-02	19.64	18026	18	153	3
GO:0009617	response to bacterium	4.76E-04	6.50E-02	4.43	18026	213	153	8
GO:0033993	response to lipid	5.05E-04	6.82E-02	3.11	18026	454	153	12
GO:0002819	regulation of adaptive immune response	5.35E-04	7.15E-02	5.94	18026	119	153	6
GO:1903034	regulation of response to wounding	5.64E-04	7.47E-02	3.54	18026	333	153	10
GO:0002831	regulation of response to biotic stimulus	5.84E-04	7.66E-02	4.91	18026	168	153	7
GO:0048584	positive regulation of response to stimulus	5.92E-04	7.69E-02	1.98	18026	1550	153	26
GO:0050704	regulation of interleukin-1 secretion	6.15E-04	7.90E-02	17.67	18026	20	153	3
GO:0031342	negative regulation of cell killing	6.15E-04	7.83E-02	17.67	18026	20	153	3
GO:0043122	regulation of I-kappaB kinase/NF-kappaB signaling	6.27E-04	7.91E-02	4.85	18026	170	153	7
GO:0032366	intracellular sterol transport	7.04E-04	8.80E-02	47.13	18026	5	153	2
GO:0034242	negative regulation of syncytium formation by plasma membrane fusion	7.04E-04	8.72E-02	47.13	18026	5	153	2
GO:0045351	type I interferon biosynthetic process	7.04E-04	8.64E-02	47.13	18026	5	153	2
GO:0050711	negative regulation of interleukin-1 secretion	7.04E-04	8.56E-02	47.13	18026	5	153	2
GO:0010575	positive regulation of vascular endothelial growth factor production	8.20E-04	9.88E-02	16.07	18026	22	153	3
GO:0002832	negative regulation of response to biotic stimulus	8.20E-04	9.79E-02	16.07	18026	22	153	3
GO:0071396	cellular response to lipid	9.31E-04	1.10E-01	3.99	18026	236	153	8
GO:0002673	regulation of acute inflammatory response	9.84E-04	1.15E-01	9.06	18026	52	153	4

Molecular functions

GO Term	Description	p-value	FDR q-value	Enrichment	N	B	n	b
GO:0003725	double-stranded RNA binding	1.25E-10	5.85E-07	18.7	18026	63	153	10
GO:0001730	2'-5'-oligoadenylate synthetase activity	5.92E-06	1.38E-02	70.69	18026	5	153	3
GO:0003950	NAD+ ADP-ribosyltransferase activity	1.09E-05	1.69E-02	27.72	18026	17	153	4
GO:0016763	transferase activity, transferring pentosyl groups	3.06E-05	3.57E-02	13.7	18026	43	153	5
GO:0017111	nucleoside-triphosphatase activity	3.49E-05	3.26E-02	3.11	18026	645	153	17
GO:0008270	zinc ion binding	6.69E-05	5.20E-02	2.64	18026	891	153	20
GO:0016462	pyrophosphatase activity	6.84E-05	4.56E-02	2.94	18026	681	153	17

GO:0016818	hydrolase activity, acting on acid anhydrides, in phosphorus-containing anhydrides	7.09E-05	4.14E-02	2.93	18026	683	153	17
GO:0003692	left-handed Z-DNA binding	7.16E-05	3.71E-02	117.82	18026	2	153	2
GO:0016817	hydrolase activity, acting on acid anhydrides	7.22E-05	3.37E-02	2.93	18026	684	153	17
GO:0046977	TAP binding	9.41E-05	3.99E-02	32.13	18026	11	153	3
GO:0097367	carbohydrate derivative binding	9.94E-05	3.86E-02	2	18026	1886	153	32
GO:0017076	purine nucleotide binding	1.12E-04	4.03E-02	2.12	18026	1558	153	28
GO:0035639	purine ribonucleoside triphosphate binding	1.70E-04	5.68E-02	2.1	18026	1513	153	27
GO:0032550	purine ribonucleoside binding	1.88E-04	5.84E-02	2.09	18026	1522	153	27
GO:0032549	ribonucleoside binding	1.94E-04	5.65E-02	2.09	18026	1525	153	27
GO:0001883	purine nucleoside binding	1.94E-04	5.32E-02	2.09	18026	1525	153	27
GO:0003723	RNA binding	1.97E-04	5.12E-02	2.16	18026	1361	153	25
GO:0003726	double-stranded RNA adenosine deaminase activity	2.14E-04	5.24E-02	78.54	18026	3	153	2
GO:0046978	TAP1 binding	2.14E-04	4.98E-02	78.54	18026	3	153	2
GO:0046979	TAP2 binding	2.14E-04	4.74E-02	78.54	18026	3	153	2
GO:0001882	nucleoside binding	2.15E-04	4.57E-02	2.07	18026	1535	153	27
GO:0042605	peptide antigen binding	2.38E-04	4.83E-02	13.09	18026	36	153	4
GO:0032555	purine ribonucleotide binding	2.49E-04	4.85E-02	2.05	18026	1549	153	27
GO:0032553	ribonucleotide binding	2.88E-04	5.37E-02	2.04	18026	1563	153	27
GO:0015248	sterol transporter activity	3.10E-04	5.55E-02	22.09	18026	16	153	3
GO:0017127	cholesterol transporter activity	3.10E-04	5.35E-02	22.09	18026	16	153	3
GO:0048020	CCR chemokine receptor binding	3.74E-04	6.22E-02	20.79	18026	17	153	3
GO:0030554	adenyl nucleotide binding	4.23E-04	6.80E-02	2.14	18026	1265	153	23
GO:0046914	transition metal ion binding	4.81E-04	7.48E-02	2.22	18026	1114	153	21
GO:0003690	double-stranded DNA binding	5.35E-04	8.05E-02	5.94	18026	119	153	6
GO:0005524	ATP binding	7.10E-04	1.03E-01	2.11	18026	1230	153	22
GO:0042379	chemokine receptor binding	7.26E-04	1.03E-01	9.82	18026	48	153	4
GO:0032559	adenyl ribonucleotide binding	9.54E-04	1.31E-01	2.06	18026	1258	153	22

Cellular component

**GO Term**	**Description**	**p-value**	**FDR q-value**	**Enrichment**	**N**	**B**	**n**	**b**
GO:0098552	side of membrane	2.61E-04	4.10E-01	3.9	18026	302	153	10
GO:0042825	TAP complex	4.25E-04	3.33E-01	58.91	18026	4	153	2

GO:0005737	cytoplasm	7.45E-04	3.89E-01	1.4	18026	5643	153	67
GO:0044217	other organism part	8.20E-04	3.21E-01	16.07	18026	22	153	3
