# Supplementary material for: Gene-Expression Profiling Suggests Impaired Signaling via the Interferon Pathway in Cstb-/- Microglia
Source: PLoS One. 2016 Jun 29;11(6):e0158195. doi: 10.1371/journal.pone.0158195 (PMC4927094; doi:10.1371/journal.pone.0158195)
Supplement: S5 Table — N = total number of genes, B = Genes associated with GO term, n = Genes at the top of the list, b = Genes at the top associated with GO term. (PDF) [file pone.0158195.s008.pdf]

Supplementary table 5: Gene ontology terms enriched in differentially expressed genes in *Cstb*$^{-/-}$ microglia identified by RNA-seq.

N = total number of genes, B = Genes associated with GO term, n = Genes at the top of the list, b = Genes at the top associated with GO term

Biological processes

GO Term	Description	p-value	FDR q-value	Enrichment	N	B	n	b
GO:0006952	defense response	3.44E-19	4.29E-15	2.48	12540	500	1212	120
GO:2000026	regulation of multicellular organismal development	1.34E-15	8.37E-12	1.97	12540	1148	928	167
GO:0009607	response to biotic stimulus	1.25E-14	5.20E-11	2.68	12540	386	1006	83
GO:0043207	response to external biotic stimulus	1.67E-14	5.20E-11	3.03	12540	368	776	69
GO:0051239	regulation of multicellular organismal process	1.67E-14	4.18E-11	1.67	12540	1674	1087	243
GO:0051707	response to other organism	2.68E-14	5.56E-11	3.01	12540	253	1006	61
GO:0009615	response to virus	4.34E-14	7.73E-11	12.97	12540	120	145	18
GO:0051240	positive regulation of multicellular organismal process	9.00E-14	1.40E-10	1.85	12540	987	1087	158
GO:0009605	response to external stimulus	1.12E-13	1.56E-10	2.03	12540	627	1214	123
GO:0050793	regulation of developmental process	1.63E-13	2.04E-10	1.95	12540	1538	557	133
GO:0006955	immune response	1.64E-13	1.86E-10	2.51	12540	388	1107	86
GO:0002376	immune system process	1.65E-13	1.71E-10	1.95	12540	811	1180	149
GO:0051704	multi-organism process	2.22E-13	2.13E-10	2.38	12540	440	1032	86
GO:0051607	defense response to virus	5.11E-13	4.55E-10	31.9	12540	94	46	11
GO:0007166	cell surface receptor signaling pathway	5.14E-13	4.28E-10	2.14	12540	972	632	105
GO:0098542	defense response to other organism	5.55E-13	4.33E-10	3.16	12540	201	1006	51
GO:0045595	regulation of cell differentiation	6.08E-13	4.46E-10	2.13	12540	1096	563	105
GO:0051094	positive regulation of developmental process	2.76E-12	1.91E-09	2.01	12540	832	863	115
GO:0050896	response to stimulus	8.75E-12	5.75E-09	1.43	12540	2501	1101	315
GO:0035456	response to interferon-beta	1.82E-11	1.14E-08	8.66	12540	28	827	16
GO:0042127	regulation of cell proliferation	1.99E-11	1.18E-08	1.83	12540	995	937	136
GO:0034097	response to cytokine	7.10E-11	4.02E-08	9.6	12540	199	105	16
GO:0002252	immune effector process	2.50E-10	1.35E-07	14.1	12540	232	46	12
GO:0002682	regulation of immune system process	2.69E-10	1.40E-07	1.79	12540	773	1180	130
GO:0051270	regulation of cellular component movement	4.61E-10	2.30E-07	2.58	12540	531	522	57
GO:0035458	cellular response to interferon-beta	7.40E-10	3.55E-07	13.72	12540	23	437	11
GO:0048525	negative regulation of viral process	7.95E-10	3.67E-07	10.31	12540	66	258	14
GO:0051960	regulation of nervous system development	1.25E-09	5.58E-07	2.38	12540	589	563	63
GO:0051241	negative regulation of multicellular organismal process	1.30E-09	5.59E-07	2.43	12540	688	458	61

GO:0045597	positive regulation of cell differentiation	1.59E-09	6.62E-07	1.84	12540	609	1233	110
GO:0022610	biological adhesion	1.75E-09	7.03E-07	2.09	12540	575	847	81
GO:0043901	negative regulation of multi-organism process	1.88E-09	7.33E-07	7.44	12540	111	258	17
GO:0048584	positive regulation of response to stimulus	2.06E-09	7.79E-07	1.57	12540	1222	1180	181
GO:2000145	regulation of cell motility	2.24E-09	8.23E-07	2.58	12540	493	522	53
GO:0045087	innate immune response	2.29E-09	8.15E-07	11.84	12540	227	56	12
GO:0040011	locomotion	2.49E-09	8.62E-07	2.07	12540	568	863	81
GO:0040012	regulation of locomotion	3.06E-09	1.03E-06	2.48	12540	543	522	56
GO:0030334	regulation of cell migration	4.06E-09	1.33E-06	2.6	12540	472	522	51
GO:0010033	response to organic substance	4.27E-09	1.36E-06	1.77	12540	951	896	120
GO:0048583	regulation of response to stimulus	4.45E-09	1.39E-06	1.46	12540	2224	874	227
GO:0006950	response to stress	4.90E-09	1.49E-06	1.47	12540	1613	1219	230
GO:0006954	inflammatory response	6.01E-09	1.79E-06	2.46	12540	209	1242	51
GO:0045071	negative regulation of viral genome replication	7.69E-09	2.23E-06	14.73	12540	33	258	10
GO:0048519	negative regulation of biological process	8.36E-09	2.37E-06	1.32	12540	3021	1134	361
GO:0050767	regulation of neurogenesis	9.68E-09	2.68E-06	2.38	12540	520	578	57
GO:0042221	response to chemical	1.00E-08	2.72E-06	1.59	12540	1226	1050	163
GO:0048518	positive regulation of biological process	1.29E-08	3.43E-06	1.26	12540	3582	1262	456
GO:0007155	cell adhesion	1.40E-08	3.64E-06	2.03	12540	569	847	78
GO:0007165	signal transduction	1.41E-08	3.58E-06	1.46	12540	2178	874	221
GO:0016477	cell migration	3.02E-08	7.54E-06	2.19	12540	463	779	63
GO:0048870	cell motility	3.14E-08	7.68E-06	2.14	12540	496	779	66
GO:0002685	regulation of leukocyte migration	4.00E-08	9.60E-06	4.71	12540	107	522	21
GO:0030595	leukocyte chemotaxis	4.49E-08	1.06E-05	4.45	12540	70	845	21
GO:0002684	positive regulation of immune system process	4.50E-08	1.04E-05	1.92	12540	453	1180	82
GO:0048523	negative regulation of cellular process	4.51E-08	1.02E-05	1.31	12540	2813	1223	359
GO:1903901	negative regulation of viral life cycle	6.31E-08	1.41E-05	9.26	12540	63	258	12
GO:0071310	cellular response to organic substance	7.45E-08	1.63E-05	1.96	12540	563	873	77
GO:0019221	cytokine-mediated signaling pathway	7.49E-08	1.61E-05	3.59	12540	146	622	26
GO:1902105	regulation of leukocyte differentiation	8.77E-08	1.85E-05	2.71	12540	159	1076	37
GO:0032879	regulation of localization	9.94E-08	2.07E-05	1.46	12540	1577	1087	200
GO:0070098	chemokine-mediated signaling pathway	1.44E-07	2.95E-05	10.01	12540	24	522	10
GO:0043900	regulation of multi-organism process	1.47E-07	2.96E-05	4.76	12540	291	172	19
GO:0060284	regulation of cell development	1.75E-07	3.46E-05	2.1	12540	639	588	63

GO:0065007	biological regulation	1.88E-07	3.66E-05	1.16	12540	6682	1012	626
GO:0008285	negative regulation of cell proliferation	1.91E-07	3.66E-05	1.89	12540	445	1179	79
GO:0050792	regulation of viral process	2.50E-07	4.72E-05	3.37	12540	114	882	27
GO:0060326	cell chemotaxis	2.56E-07	4.77E-05	4.45	12540	108	522	20
GO:0006935	chemotaxis	2.61E-07	4.79E-05	2.85	12540	171	850	33
GO:0035455	response to interferon-alpha	2.64E-07	4.78E-05	9.89	12540	15	761	9
GO:0001817	regulation of cytokine production	2.67E-07	4.75E-05	2.01	12540	340	1193	65
GO:0042330	taxis	2.82E-07	4.96E-05	2.83	12540	172	850	33
GO:0050789	regulation of biological process	2.91E-07	5.05E-05	1.17	12540	6418	1012	604
GO:0002687	positive regulation of leukocyte migration	3.96E-07	6.76E-05	5.04	12540	81	522	17
GO:0048522	positive regulation of cellular process	5.02E-07	8.45E-05	1.26	12540	3191	1262	404
GO:0045664	regulation of neuron differentiation	5.54E-07	9.22E-05	2.38	12540	425	557	45
GO:0006928	movement of cell or subcellular component	5.55E-07	9.10E-05	1.7	12540	728	1045	103
GO:0048585	negative regulation of response to stimulus	8.25E-07	1.34E-04	1.68	12540	899	873	105
GO:0051093	negative regulation of developmental process	8.29E-07	1.33E-04	1.69	12540	620	1211	101
GO:1903706	regulation of hemopoiesis	8.66E-07	1.37E-04	2.31	12540	222	1076	44
GO:0045069	regulation of viral genome replication	9.95E-07	1.55E-04	9.35	12540	52	258	10
GO:0051962	positive regulation of nervous system development	1.36E-06	2.09E-04	2.06	12540	367	928	56
GO:0070887	cellular response to chemical stimulus	1.40E-06	2.13E-04	1.75	12540	721	873	88
GO:0071345	cellular response to cytokine stimulus	1.43E-06	2.14E-04	3.28	12540	115	830	25
GO:2000147	positive regulation of cell motility	1.43E-06	2.12E-04	2.1	12540	285	1087	52
GO:0023056	positive regulation of signaling	1.45E-06	2.13E-04	1.64	12540	964	874	110
GO:0050900	leukocyte migration	1.67E-06	2.43E-04	3.45	12540	99	845	23
GO:0008284	positive regulation of cell proliferation	2.21E-06	3.16E-04	1.83	12540	552	931	75
GO:0097530	granulocyte migration	2.28E-06	3.24E-04	5.07	12540	41	845	14
GO:0035457	cellular response to interferon-alpha	2.35E-06	3.29E-04	180.87	12540	8	26	3
GO:0010647	positive regulation of cell communication	2.56E-06	3.55E-04	1.59	12540	1055	874	117
GO:0043903	regulation of symbiosis, encompassing mutualism through parasitism	2.71E-06	3.71E-04	4.01	12540	136	414	18
GO:0040017	positive regulation of locomotion	2.72E-06	3.69E-04	2.05	12540	298	1087	53
GO:0009617	response to bacterium	2.96E-06	3.98E-04	2.7	12540	122	1180	31
GO:0050776	regulation of immune response	3.45E-06	4.57E-04	1.93	12540	330	1180	60
GO:0051272	positive regulation of cellular component movement	3.73E-06	4.89E-04	2.05	12540	293	1087	52
GO:1903900	regulation of viral life cycle	3.96E-06	5.14E-04	3.19	12540	107	882	24

GO:0040013	negative regulation of locomotion	4.40E-06	5.65E-04	3.22	12540	183	490	23
GO:0070208	protein heterotrimerization	4.45E-06	5.66E-04	25.68	12540	11	222	5
GO:0031347	regulation of defense response	4.67E-06	5.88E-04	1.87	12540	363	1180	64
GO:0032101	regulation of response to external stimulus	4.69E-06	5.85E-04	1.81	12540	560	904	73
GO:0050794	regulation of cellular process	4.79E-06	5.92E-04	1.16	12540	6130	1012	573
GO:0030336	negative regulation of cell migration	5.44E-06	6.65E-04	3.48	12540	147	490	20
GO:0030335	positive regulation of cell migration	5.46E-06	6.61E-04	2.05	12540	281	1087	50
GO:0033993	response to lipid	6.01E-06	7.21E-04	2.1	12540	320	896	48
GO:0097529	myeloid leukocyte migration	7.16E-06	8.51E-04	4.17	12540	57	845	16
GO:0050795	regulation of behavior	7.27E-06	8.56E-04	2.3	12540	156	1259	36
GO:2000401	regulation of lymphocyte migration	7.55E-06	8.80E-04	17.56	12540	28	153	6
GO:0051049	regulation of transport	7.59E-06	8.77E-04	1.47	12540	1143	1094	147
GO:0071621	granulocyte chemotaxis	7.84E-06	8.97E-04	4.95	12540	39	845	13
GO:0045596	negative regulation of cell differentiation	8.24E-06	9.34E-04	1.72	12540	480	1197	79
GO:0048520	positive regulation of behavior	9.07E-06	1.02E-03	2.71	12540	103	1259	28
GO:0050768	negative regulation of neurogenesis	9.30E-06	1.04E-03	3.3	12540	192	416	21
GO:0032496	response to lipopolysaccharide	9.75E-06	1.08E-03	2.7	12540	140	896	27
GO:0051271	negative regulation of cellular component movement	9.81E-06	1.07E-03	3.26	12540	165	490	21
GO:0051961	negative regulation of nervous system development	1.04E-05	1.13E-03	3.19	12540	208	416	22
GO:0042742	defense response to bacterium	1.05E-05	1.13E-03	2.76	12540	104	1180	27
GO:0009967	positive regulation of signal transduction	1.12E-05	1.20E-03	1.63	12540	856	874	97
GO:2000146	negative regulation of cell motility	1.24E-05	1.31E-03	3.35	12540	153	490	20
GO:1990266	neutrophil migration	1.35E-05	1.42E-03	5.09	12540	35	845	12
GO:0050920	regulation of chemotaxis	1.53E-05	1.59E-03	2.98	12540	117	862	24
GO:0022603	regulation of anatomical structure morphogenesis	1.67E-05	1.72E-03	2.04	12540	638	473	49
GO:1901342	regulation of vasculature development	1.68E-05	1.72E-03	3.14	12540	151	556	21
GO:0023051	regulation of signaling	1.83E-05	1.86E-03	1.41	12540	1825	808	166
GO:0010646	regulation of cell communication	1.94E-05	1.95E-03	1.38	12540	1935	874	186
GO:0044707	single-multicellular organism process	1.97E-05	1.97E-03	1.36	12540	1723	1099	205
GO:0003008	system process	2.07E-05	2.05E-03	1.69	12540	571	1025	79
GO:0009966	regulation of signal transduction	2.07E-05	2.03E-03	1.42	12540	1615	874	160
GO:0002237	response to molecule of bacterial origin	2.14E-05	2.09E-03	2.99	12540	155	596	22
GO:0090090	negative regulation of canonical Wnt signaling pathway	2.28E-05	2.21E-03	4.14	12540	67	678	15
GO:0030111	regulation of Wnt signaling pathway	2.32E-05	2.23E-03	2.89	12540	159	628	23

GO:0010648	negative regulation of cell communication	2.47E-05	2.35E-03	1.63	12540	782	873	89
GO:0045785	positive regulation of cell adhesion	2.55E-05	2.41E-03	2.01	12540	239	1176	45
GO:0030155	regulation of cell adhesion	2.62E-05	2.45E-03	1.74	12540	422	1176	69
GO:0050890	cognition	2.71E-05	2.53E-03	2.31	12540	175	1025	33
GO:0002028	regulation of sodium ion transport	2.79E-05	2.58E-03	5.66	12540	54	451	11
GO:0001819	positive regulation of cytokine production	2.83E-05	2.59E-03	2.05	12540	215	1193	42
GO:0032501	multicellular organismal process	2.83E-05	2.58E-03	1.35	12540	1733	1099	205
GO:2000404	regulation of T cell migration	3.05E-05	2.75E-03	19.51	12540	21	153	5
GO:0008202	steroid metabolic process	3.13E-05	2.81E-03	2.51	12540	119	1219	29
GO:0023057	negative regulation of signaling	3.14E-05	2.80E-03	1.63	12540	776	873	88
GO:0030178	negative regulation of Wnt signaling pathway	3.33E-05	2.94E-03	3.66	12540	86	678	17
GO:0002688	regulation of leukocyte chemotaxis	3.41E-05	3.00E-03	4.66	12540	67	522	13
GO:0045665	negative regulation of neuron differentiation	3.45E-05	3.01E-03	3.53	12540	145	416	17
GO:0050921	positive regulation of chemotaxis	3.54E-05	3.07E-03	2.75	12540	87	1259	24
GO:0016125	sterol metabolic process	3.86E-05	3.32E-03	2.96	12540	73	1219	21
GO:0002828	regulation of type 2 immune response	4.46E-05	3.81E-03	5.87	12540	20	961	9
GO:0014015	positive regulation of gliogenesis	4.48E-05	3.80E-03	4.01	12540	48	911	14
GO:0001911	negative regulation of leukocyte mediated cytotoxicity	4.55E-05	3.83E-03	29.23	12540	13	132	4
GO:0031342	negative regulation of cell killing	4.55E-05	3.81E-03	29.23	12540	13	132	4
GO:0030593	neutrophil chemotaxis	4.70E-05	3.91E-03	4.95	12540	33	845	11
GO:0050877	neurological system process	5.19E-05	4.29E-03	1.8	12540	401	1025	59
GO:0080134	regulation of response to stress	5.29E-05	4.34E-03	1.49	12540	848	1183	119
GO:0045765	regulation of angiogenesis	5.43E-05	4.43E-03	3.08	12540	139	556	19
GO:0050769	positive regulation of neurogenesis	6.08E-05	4.92E-03	2.44	12540	315	473	29
GO:0071347	cellular response to interleukin-1	6.49E-05	5.22E-03	8.31	12540	19	556	7
GO:0010817	regulation of hormone levels	6.90E-05	5.51E-03	2.58	12540	119	1061	26
GO:0034340	response to type I interferon	6.94E-05	5.52E-03	119.43	12540	2	105	2
GO:0010959	regulation of metal ion transport	7.29E-05	5.76E-03	2.81	12540	216	454	22
GO:0002683	negative regulation of immune system process	8.13E-05	6.37E-03	2.04	12540	265	927	40
GO:0008203	cholesterol metabolic process	8.86E-05	6.90E-03	3.29	12540	66	983	17
GO:0055065	metal ion homeostasis	8.98E-05	6.96E-03	2.43	12540	267	542	28
GO:0043271	negative regulation of ion transport	9.62E-05	7.41E-03	4.64	12540	78	416	12
GO:1901701	cellular response to oxygen-containing compound	9.99E-05	7.64E-03	1.91	12540	354	873	47
GO:2000402	negative regulation of lymphocyte migration	1.09E-04	8.29E-03	152	12540	3	55	2

GO:0007611	learning or memory	1.15E-04	8.72E-03	2.27	12540	156	1025	29
GO:0010721	negative regulation of cell development	1.21E-04	9.11E-03	2.28	12540	226	731	30
GO:0002690	positive regulation of leukocyte chemotaxis	1.22E-04	9.13E-03	4.8	12540	55	522	11
GO:0010720	positive regulation of cell development	1.26E-04	9.32E-03	1.86	12540	382	863	49
GO:0055082	cellular chemical homeostasis	1.31E-04	9.63E-03	2.05	12540	291	798	38
GO:0042590	antigen processing and presentation of exogenous peptide antigen via MHC class I	1.37E-04	1.00E-02	9.02	12540	6	1158	5
GO:0000902	cell morphogenesis	1.39E-04	1.02E-02	2.58	12540	164	681	23
GO:0019934	cGMP-mediated signaling	1.45E-04	1.05E-02	8.89	12540	6	1176	5
GO:0043269	regulation of ion transport	1.52E-04	1.10E-02	2.29	12540	362	454	30
GO:0044708	single-organism behavior	1.56E-04	1.12E-02	1.84	12540	284	1152	48
GO:0030199	collagen fibril organization	1.66E-04	1.19E-02	52.25	12540	24	30	3
GO:0030003	cellular cation homeostasis	1.74E-04	1.23E-02	2.03	12540	256	894	37
GO:0045088	regulation of innate immune response	1.75E-04	1.23E-02	6.21	12540	122	149	9
GO:0030198	extracellular matrix organization	1.86E-04	1.30E-02	2.56	12540	121	972	24
GO:1902107	positive regulation of leukocyte differentiation	1.91E-04	1.33E-02	2.72	12540	90	1076	21
GO:0051130	positive regulation of cellular component organization	1.93E-04	1.34E-02	1.53	12540	901	867	95
GO:0015804	neutral amino acid transport	1.94E-04	1.34E-02	9.4	12540	23	348	6
GO:0032103	positive regulation of response to external stimulus	1.97E-04	1.35E-02	2.07	12540	267	793	35
GO:0006875	cellular metal ion homeostasis	2.07E-04	1.41E-02	2.17	12540	224	798	31
GO:0050865	regulation of cell activation	2.08E-04	1.41E-02	2.32	12540	304	497	28
GO:0043062	extracellular structure organization	2.19E-04	1.47E-02	2.54	12540	122	972	24
GO:0051216	cartilage development	2.27E-04	1.53E-02	209	12540	60	2	2
GO:1903708	positive regulation of hemopoiesis	2.44E-04	1.63E-02	2.5	12540	112	1076	24
GO:0050870	positive regulation of T cell activation	2.56E-04	1.70E-02	3.69	12540	101	471	14
GO:0032020	ISG15-protein conjugation	2.66E-04	1.76E-02	28.07	12540	5	268	3
GO:1903792	negative regulation of anion transport	2.78E-04	1.83E-02	6.98	12540	25	503	7
GO:0098602	single organism cell adhesion	2.79E-04	1.82E-02	1.82	12540	300	1082	47
GO:0006873	cellular ion homeostasis	2.79E-04	1.81E-02	1.99	12540	261	894	37
GO:1901699	cellular response to nitrogen compound	2.86E-04	1.85E-02	2.77	12540	188	458	19
GO:0035929	steroid hormone secretion	2.87E-04	1.84E-02	93.93	12540	3	89	2
GO:0051128	regulation of cellular component organization	2.89E-04	1.85E-02	1.36	12540	1657	873	157
GO:0006721	terpenoid metabolic process	2.90E-04	1.84E-02	4.41	12540	29	980	10
GO:0050778	positive regulation of immune response	2.91E-04	1.84E-02	1.89	12540	231	1180	41

GO:0015825	L-serine transport	2.91E-04	1.83E-02	27.26	12540	5	276	3
GO:0001503	ossification	2.96E-04	1.86E-02	6.45	12540	71	219	8
GO:0042445	hormone metabolic process	2.97E-04	1.85E-02	3	12540	67	1061	17
GO:0001910	regulation of leukocyte mediated cytotoxicity	3.07E-04	1.90E-02	12.84	12540	37	132	5
GO:0071417	cellular response to organonitrogen compound	3.13E-04	1.93E-02	2.85	12540	173	458	18
GO:0014013	regulation of gliogenesis	3.13E-04	1.93E-02	2.91	12540	85	911	18
GO:0070206	protein trimerization	3.19E-04	1.95E-02	8.93	12540	30	281	6
GO:0031349	positive regulation of defense response	3.38E-04	2.06E-02	2.11	12540	146	1180	29
GO:0071222	cellular response to lipopolysaccharide	3.38E-04	2.05E-02	3.3	12540	69	827	15
GO:0018149	peptide cross-linking	3.38E-04	2.04E-02	17.71	12540	16	177	4
GO:1902930	regulation of alcohol biosynthetic process	3.41E-04	2.04E-02	3.74	12540	39	1032	12
GO:0009719	response to endogenous stimulus	3.43E-04	2.05E-02	1.63	12540	506	1017	67
GO:1902993	positive regulation of amyloid precursor protein catabolic process	3.46E-04	2.05E-02	23.17	12540	4	406	3
GO:1902004	positive regulation of beta-amyloid formation	3.46E-04	2.04E-02	23.17	12540	4	406	3
GO:0002716	negative regulation of natural killer cell mediated immunity	3.48E-04	2.05E-02	33.86	12540	11	101	3
GO:0045953	negative regulation of natural killer cell mediated cytotoxicity	3.48E-04	2.04E-02	33.86	12540	11	101	3
GO:0051050	positive regulation of transport	3.68E-04	2.14E-02	1.54	12540	626	1094	84
GO:0019731	antibacterial humoral response	3.71E-04	2.15E-02	7.37	12540	14	729	6
GO:0009968	negative regulation of signal transduction	3.75E-04	2.17E-02	1.57	12540	703	873	77
GO:0098771	inorganic ion homeostasis	3.86E-04	2.22E-02	1.87	12540	315	894	42
GO:1901700	response to oxygen-containing compound	3.87E-04	2.22E-02	1.61	12540	608	896	70
GO:0009611	response to wounding	3.88E-04	2.21E-02	5.44	12540	85	244	9
GO:0007162	negative regulation of cell adhesion	3.90E-04	2.21E-02	2.12	12540	158	1087	29
GO:0055074	calcium ion homeostasis	3.92E-04	2.21E-02	2.61	12540	177	542	20
GO:2000242	negative regulation of reproductive process	3.93E-04	2.21E-02	160.77	12540	26	6	2
GO:0034112	positive regulation of homotypic cell-cell adhesion	3.98E-04	2.23E-02	3.55	12540	105	471	14
GO:1903039	positive regulation of leukocyte cell-cell adhesion	3.98E-04	2.22E-02	3.55	12540	105	471	14
GO:0032608	interferon-beta production	3.99E-04	2.21E-02	79.62	12540	3	105	2
GO:0055080	cation homeostasis	4.09E-04	2.26E-02	1.89	12540	305	894	41
GO:0022407	regulation of cell-cell adhesion	4.13E-04	2.27E-02	1.9	12540	237	1087	39
GO:0051249	regulation of lymphocyte activation	4.13E-04	2.26E-02	1.9	12540	237	1087	39
GO:0034341	response to interferon-gamma	4.18E-04	2.28E-02	4.28	12540	32	916	10

GO:0048246	macrophage chemotaxis	4.24E-04	2.30E-02	30.07	12540	9	139	3
GO:0033159	negative regulation of protein import into nucleus, translocation	4.30E-04	2.32E-02	16.48	12540	3	761	3
GO:0048247	lymphocyte chemotaxis	4.37E-04	2.35E-02	31.99	12540	12	98	3
GO:2000551	regulation of T-helper 2 cell cytokine production	4.37E-04	2.34E-02	16.37	12540	3	766	3
GO:0045472	response to ether	4.41E-04	2.35E-02	59.15	12540	2	212	2
GO:0019882	antigen processing and presentation	4.43E-04	2.35E-02	2.88	12540	64	1158	17
GO:0002703	regulation of leukocyte mediated immunity	4.46E-04	2.36E-02	2.41	12540	114	1097	24
GO:2000739	regulation of mesenchymal stem cell differentiation	4.53E-04	2.39E-02	11.6	12540	6	721	4
GO:0090025	regulation of monocyte chemotaxis	4.55E-04	2.38E-02	5.55	12540	14	1129	7
GO:1902003	regulation of beta-amyloid formation	4.66E-04	2.43E-02	13.73	12540	9	406	4
GO:0051046	regulation of secretion	4.71E-04	2.45E-02	2	12540	439	514	36
GO:0044406	adhesion of symbiont to host	4.77E-04	2.47E-02	8.42	12540	9	827	5
GO:0007167	enzyme linked receptor protein signaling pathway	4.82E-04	2.48E-02	1.93	12540	323	763	38
GO:0048731	system development	4.86E-04	2.50E-02	1.76	12540	458	795	51
GO:0019369	arachidonic acid metabolic process	4.89E-04	2.50E-02	4.94	12540	19	1069	8
GO:1903530	regulation of secretion by cell	4.97E-04	2.53E-02	2.03	12540	408	514	34
GO:0051251	positive regulation of lymphocyte activation	4.98E-04	2.52E-02	2.1	12540	148	1129	28
GO:0090026	positive regulation of monocyte chemotaxis	5.02E-04	2.53E-02	14.29	12540	11	319	4
GO:0061326	renal tubule development	5.06E-04	2.54E-02	97.21	12540	6	43	2
GO:0071396	cellular response to lipid	5.09E-04	2.55E-02	2.24	12540	162	862	25
GO:0034754	cellular hormone metabolic process	5.09E-04	2.54E-02	3.62	12540	41	1014	12
GO:0060828	regulation of canonical Wnt signaling pathway	5.23E-04	2.60E-02	2.87	12540	116	678	18
GO:0031341	regulation of cell killing	5.26E-04	2.60E-02	11.59	12540	41	132	5
GO:1903522	regulation of blood circulation	5.38E-04	2.65E-02	2.37	12540	114	1112	24
GO:0010942	positive regulation of cell death	5.41E-04	2.66E-02	1.68	12540	463	937	58
GO:0050801	ion homeostasis	5.45E-04	2.66E-02	1.83	12540	329	894	43
GO:0006874	cellular calcium ion homeostasis	5.57E-04	2.71E-02	2.6	12540	169	542	19
GO:0044057	regulation of system process	5.63E-04	2.73E-02	1.82	12540	256	1129	42
GO:0072503	cellular divalent inorganic cation homeostasis	5.71E-04	2.76E-02	2.54	12540	182	542	20
GO:0019730	antimicrobial humoral response	5.84E-04	2.81E-02	6.88	12540	15	729	6
GO:0002830	positive regulation of type 2 immune response	5.86E-04	2.81E-02	12.81	12540	9	435	4
GO:0034446	substrate adhesion-dependent cell spreading	5.86E-04	2.80E-02	6.14	12540	25	572	7
GO:1901623	regulation of lymphocyte chemotaxis	5.88E-04	2.80E-02	29.53	12540	13	98	3

GO:0032329	serine transport	5.88E-04	2.79E-02	22.72	12540	6	276	3
GO:0071346	cellular response to interferon-gamma	5.90E-04	2.79E-02	5.64	12540	17	916	7
GO:1902563	regulation of neutrophil activation	5.93E-04	2.79E-02	9.72	12540	5	1032	4
GO:0043270	positive regulation of ion transport	5.99E-04	2.81E-02	2.87	12540	155	451	16
GO:0072507	divalent inorganic cation homeostasis	6.04E-04	2.82E-02	2.47	12540	197	542	21
GO:0050867	positive regulation of cell activation	6.52E-04	3.03E-02	2.66	12540	178	476	18
GO:0034367	macromolecular complex remodeling	6.60E-04	3.06E-02	7.8	12540	9	893	5
GO:0034368	protein-lipid complex remodeling	6.60E-04	3.05E-02	7.8	12540	9	893	5
GO:0034369	plasma lipoprotein particle remodeling	6.60E-04	3.04E-02	7.8	12540	9	893	5
GO:0010243	response to organonitrogen compound	6.94E-04	3.18E-02	2.27	12540	301	458	25
GO:0001957	intramembranous ossification	6.96E-04	3.18E-02	4 180.00	12540	3	1	1
GO:0036072	direct ossification	6.96E-04	3.17E-02	4 180.00	12540	3	1	1
GO:0002474	antigen processing and presentation of peptide antigen via MHC class I	7.03E-04	3.19E-02	4.24	12540	23	1158	9
GO:0060340	positive regulation of type I interferon-mediated signaling pathway	7.12E-04	3.21E-02	89.57	12540	7	40	2
GO:0043588	skin development	7.54E-04	3.39E-02	119.43	12540	30	7	2
GO:0034110	regulation of homotypic cell-cell adhesion	7.98E-04	3.58E-02	1.95	12540	182	1129	32
GO:0060371	regulation of atrial cardiac muscle cell membrane depolarization	8.26E-04	3.69E-02	65.31	12540	3	128	2
GO:1902533	positive regulation of intracellular signal transduction	8.61E-04	3.83E-02	1.52	12540	568	1148	79
GO:0006720	isoprenoid metabolic process	9.25E-04	4.10E-02	3.41	12540	45	980	12
GO:0044087	regulation of cellular component biogenesis	9.28E-04	4.10E-02	2.12	12540	505	340	29
GO:0001568	blood vessel development	9.30E-04	4.10E-02	3.86	12540	63	567	11
GO:0043589	skin morphogenesis	9.36E-04	4.11E-02	83.6	12540	10	30	2
GO:0048002	antigen processing and presentation of peptide antigen	9.47E-04	4.14E-02	3.33	12540	39	1158	12
GO:0034240	negative regulation of macrophage fusion	9.57E-04	4.17E-02	1 045.00	12540	1	12	1
GO:0006695	cholesterol biosynthetic process	9.62E-04	4.18E-02	4.09	12540	24	1150	9
GO:1903034	regulation of response to wounding	9.69E-04	4.20E-02	1.92	12540	247	897	34
GO:0016126	sterol biosynthetic process	9.87E-04	4.26E-02	4.23	12540	29	920	9
GO:0032332	positive regulation of chondrocyte differentiation	9.88E-04	4.25E-02	11.04	12540	9	505	4
GO:0071495	cellular response to endogenous stimulus	9.89E-04	4.24E-02	2.15	12540	311	506	27

Molecular functions

GO Term	Description	p-value	FDR q-value	Enrichment	N	B	n	b
GO:0042379	chemokine receptor binding	5.77E-08	2.49E-04	7.45	12540	35	625	13
GO:0005539	glycosaminoglycan binding	1.43E-07	3.09E-04	4.41	12540	115	519	21
GO:0008009	chemokine activity	1.56E-07	2.25E-04	8.49	12540	26	625	11
GO:0048020	CCR chemokine receptor binding	7.52E-07	8.12E-04	39.99	12540	16	98	5
GO:0005125	cytokine activity	8.29E-07	7.16E-04	3.73	12540	95	779	22
GO:0005515	protein binding	9.33E-07	6.71E-04	1.16	12540	5561	1262	651
GO:0048407	platelet-derived growth factor binding	1.78E-06	1.10E-03	29.16	12540	10	215	5
GO:0005102	receptor binding	1.89E-06	1.02E-03	1.67	12540	912	831	101
GO:0008201	heparin binding	3.03E-06	1.46E-03	4.66	12540	83	519	16
GO:0005126	cytokine receptor binding	3.56E-06	1.54E-03	3.04	12540	165	625	25
GO:0004872	receptor activity	9.66E-06	3.79E-03	2.09	12540	528	545	48
GO:1901681	sulfur compound binding	1.38E-05	4.95E-03	3.1	12540	157	567	22
GO:0060089	molecular transducer activity	1.56E-05	5.17E-03	1.91	12540	699	545	58
GO:0038023	signaling receptor activity	2.35E-05	7.26E-03	2.14	12540	436	564	42
GO:0004871	signal transducer activity	4.37E-05	1.26E-02	1.93	12540	608	545	51
GO:0004888	transmembrane signaling receptor activity	4.92E-05	1.33E-02	2.22	12540	378	537	36
GO:0005201	extracellular matrix structural constituent	5.11E-05	1.30E-02	73.76	12540	17	30	3
GO:0015175	neutral amino acid transmembrane transporter activity	7.77E-05	1.86E-02	10.81	12540	20	348	6
GO:0048248	CXCR3 chemokine receptor binding	8.53E-05	1.94E-02	28.31	12540	3	443	3
GO:0019838	growth factor binding	1.11E-04	2.39E-02	5.1	12540	92	294	11
GO:0045236	CXCR chemokine receptor binding	1.21E-04	2.50E-02	12.01	12540	10	522	5
GO:0001664	G-protein coupled receptor binding	1.44E-04	2.82E-02	3.02	12540	161	465	18
GO:0022889	serine transmembrane transporter activity	2.91E-04	5.46E-02	27.26	12540	5	276	3
GO:0015194	L-serine transmembrane transporter activity	2.91E-04	5.24E-02	27.26	12540	5	276	3
GO:0031727	CCR2 chemokine receptor binding	4.33E-04	7.47E-02	90.87	12540	4	69	2
GO:0004896	cytokine receptor activity	4.81E-04	8.00E-02	8.84	12540	56	152	6
GO:0070888	E-box binding	5.47E-04	8.75E-02	5.86	12540	19	788	7
GO:0000982	RNA polymerase II core promoter proximal region sequence-specific DNA binding transcription factor activity	7.29E-04	1.12E-01	1.89	12540	222	1074	36
GO:0019770	IgG receptor activity	7.98E-04	1.19E-01	15.05	12540	3	833	3
GO:0016493	C-C chemokine receptor activity	8.43E-04	1.21E-01	21.41	12540	7	251	3

GO:0000979	RNA polymerase II core promoter sequence-specific DNA binding	8.66E-04	1.21E-01	4.03	12540	39	797	10
GO:0001730	2'-5'-oligoadenylate synthetase activity	9.42E-04	1.27E-01	18.17	12540	5	414	3

Cellular component

GO Term	Description	p-value	FDR q-value	Enrichment	N	B	n	b
GO:0005615	extracellular space	1.35E-19	2.04E-16	2.72	12540	650	767	108
GO:0005576	extracellular region	3.15E-18	2.37E-15	2.9	12540	761	523	92
GO:0031012	extracellular matrix	4.64E-13	2.33E-10	5.55	12540	217	302	29
GO:0044459	plasma membrane part	2.32E-11	8.76E-09	1.64	12540	1197	1194	187
GO:0005578	proteinaceous extracellular matrix	2.70E-11	8.14E-09	5.79	12540	172	302	24
GO:0005886	plasma membrane	8.56E-10	2.15E-07	1.4	12540	2169	1261	305
GO:0044421	extracellular region part	1.48E-09	3.18E-07	1.47	12540	2472	785	228
GO:0005581	collagen trimer	1.41E-08	2.65E-06	11.97	12540	45	256	11
GO:0044425	membrane part	4.55E-08	7.62E-06	1.27	12540	3640	1132	417
GO:0097458	neuron part	7.82E-08	1.18E-05	1.57	12540	963	1261	152
GO:0031224	intrinsic component of membrane	1.24E-07	1.70E-05	1.3	12540	2778	1248	359
GO:0098552	side of membrane	2.85E-07	3.58E-05	2.44	12540	197	1097	42
GO:0005583	fibrillar collagen trimer	1.98E-06	2.30E-04	179.14	12540	7	30	3
GO:0016021	integral component of membrane	2.44E-06	2.62E-04	1.27	12540	2688	1248	341
GO:0009897	external side of plasma membrane	3.16E-06	3.17E-04	2.48	12540	157	1097	34
GO:0044420	extracellular matrix component	3.77E-06	3.55E-04	5.87	12540	92	302	13
GO:0098589	membrane region	7.90E-06	7.00E-04	1.59	12540	677	1248	107
GO:0005584	collagen type I trimer	1.11E-05	9.26E-04	418	12540	2	30	2
GO:0016020	membrane	1.43E-05	1.14E-03	1.16	12540	5347	1152	570
GO:0031226	intrinsic component of plasma membrane	1.83E-05	1.38E-03	1.98	12540	400	808	51
GO:0030426	growth cone	3.29E-05	2.36E-03	4.86	12540	114	294	13
GO:0030427	site of polarized growth	4.66E-05	3.20E-03	4.74	12540	117	294	13
GO:0043005	neuron projection	4.72E-05	3.10E-03	1.58	12540	668	1103	93
GO:0045121	membrane raft	5.97E-05	3.75E-03	1.97	12540	219	1248	43
GO:0005887	integral component of plasma membrane	6.92E-05	4.17E-03	1.96	12540	365	808	46
GO:0042995	cell projection	1.44E-04	8.34E-03	1.38	12540	1191	1217	159
GO:0044217	other organism part	3.15E-04	1.76E-02	6.24	12540	17	827	7

GO:1990777	lipoprotein particle	3.36E-04	1.81E-02	38.82	12540	19	51	3
GO:0034358	plasma lipoprotein particle	3.36E-04	1.75E-02	38.82	12540	19	51	3
GO:0030425	dendrite	3.49E-04	1.75E-02	1.98	12540	278	845	37
GO:0031225	anchored component of membrane	3.73E-04	1.81E-02	3.5	12540	83	604	14
GO:0030424	axon	4.47E-04	2.10E-02	1.89	12540	239	1082	39
GO:0032994	protein-lipid complex	4.86E-04	2.22E-02	35.13	12540	21	51	3
GO:0005586	collagen type III trimer	5.58E-04	2.47E-02	1 791.43	12540	1	7	1
GO:0072562	blood microparticle	5.81E-04	2.50E-02	3.5	12540	65	717	13
GO:0009986	cell surface	6.01E-04	2.51E-02	1.63	12540	369	1248	60
GO:0020005	symbiont-containing vacuole membrane	7.28E-04	2.97E-02	10.11	12540	6	827	4
GO:0033267	axon part	7.50E-04	2.98E-02	3.53	12540	176	242	12
GO:0033643	host cell part	9.12E-04	3.52E-02	7.58	12540	10	827	5
