# Supplementary material for: Gene-Expression Profiling Suggests Impaired Signaling via the Interferon Pathway in Cstb-/- Microglia
Source: PLoS One. 2016 Jun 29;11(6):e0158195. doi: 10.1371/journal.pone.0158195 (PMC4927094; doi:10.1371/journal.pone.0158195)
Supplement: S6 Table — (PDF) [file pone.0158195.s009.pdf]

Supplementary table 6: Pathways enriched in differentially expressed genes in *Cstb* $^{-/-}$ microglia.

**Pathways identified in microarray approach**	**-log(p-value)**
Activation of IRF by Cytosolic Pattern Recognition Receptors	1.25E+01
Interferon Signaling	1.04E+01
Role of Pattern Recognition Receptors in Recognition of Bacteria and Viruses	8.02E+00
Role of RIG1-like Receptors in Antiviral Innate Immunity	6.38E+00
Antigen Presentation Pathway	5.42E+00
Role of PKR in Interferon Induction and Antiviral Response	5.25E+00
Type I Diabetes Mellitus Signaling	5.15E+00
Communication between Innate and Adaptive Immune Cells	4.57E+00
Retinoic acid Mediated Apoptosis Signaling	4.24E+00
Dendritic Cell Maturation	3.79E+00
Graft-versus-Host Disease Signaling	3.59E+00
Regulation of Cytokine Production in Intestinal Epithelial Cells by IL-17A and IL-17F	3.38E+00
LXR/RXR Activation	2.95E+00
Pathogenesis of Multiple Sclerosis	2.84E+00
Allograft Rejection Signaling	2.64E+00
UVA-Induced MAPK Signaling	2.60E+00
iNOS Signaling	2.55E+00
Death Receptor Signaling	2.53E+00
Role of Hypercytokinemia/hyperchemokinemia in the Pathogenesis of Influenza	2.52E+00
Systemic Lupus Erythematosus Signaling	2.52E+00
Autoimmune Thyroid Disease Signaling	2.41E+00
CDP-diacylglycerol Biosynthesis I	2.33E+00
B Cell Receptor Signaling	2.25E+00
Regulation of Cytokine Production in Macrophages and T Helper Cells by IL-17A and IL-17F	2.23E+00
Phosphatidylglycerol Biosynthesis II (Non-plastidic)	2.23E+00
Role of JAK1, JAK2 and TYK2 in Interferon Signaling	1.99E+00
IL-17A Signaling in Gastric Cells	1.95E+00
IL-12 Signaling and Production in Macrophages	1.95E+00
Toll-like Receptor Signaling	1.93E+00
TREM1 Signaling	1.92E+00
FLT3 Signaling in Hematopoietic Progenitor Cells	1.92E+00
IL-15 Production	1.89E+00
VDR/RXR Activation	1.86E+00
TNFR2 Signaling	1.83E+00
Crosstalk between Dendritic Cells and Natural Killer Cells	1.70E+00
OX40 Signaling Pathway	1.70E+00
IL-17A Signaling in Fibroblasts	1.67E+00
Salvage Pathways of Pyrimidine Ribonucleotides	1.65E+00
Acute Phase Response Signaling	1.62E+00
Role of NFAT in Regulation of the Immune Response	1.61E+00
NF-kB Signaling	1.59E+00
Thyroid Cancer Signaling	1.56E+00

Neuroprotective Role of THOP1 in Alzheimer's Disease	1.56E+00
Granulocyte Adhesion and Diapedesis	1.56E+00
PPARa/RXRa Activation	1.54E+00
Production of Nitric Oxide and Reactive Oxygen Species in Macrophages	1.54E+00
Role of IL-17F in Allergic Inflammatory Airway Diseases	1.49E+00
Glucocorticoid Receptor Signaling	1.49E+00
NAD Biosynthesis III	1.42E+00
TNFR1 Signaling	1.40E+00
PKd Signaling in T Lymphocytes	1.39E+00
phagosome maturation	1.37E+00
CD27 Signaling in Lymphocytes	1.35E+00
Primary Immunodeficiency Signaling	1.35E+00
PI3K/AKT Signaling	1.35E+00
Thrombopoietin Signaling	1.31E+00
PI3K Signaling in B Lymphocytes	1.30E+00
Role of JAK1 and JAK3 in gc Cytokine Signaling	1.21E+00
Pyridoxal 5'-phosphate Salvage Pathway	1.19E+00
CD40 Signaling	1.18E+00
IL-10 Signaling	1.15E+00
Role of MAPK Signaling in the Pathogenesis of Influenza	1.14E+00
IL-3 Signaling	1.12E+00
T Helper Cell Differentiation	1.12E+00
Small Cell Lung Cancer Signaling	1.12E+00
JAK/Stat Signaling	1.10E+00
NF-kB Activation by Viruses	1.09E+00
Prolactin Signaling	1.09E+00
NAD Phosphorylation and Dephosphorylation	1.09E+00
Protein Ubiquitination Pathway	1.08E+00
G-Protein Coupled Receptor Signaling	1.08E+00
PDGF Signaling	1.06E+00
Cdc42 Signaling	1.03E+00
CTLA4 Signaling in Cytotoxic T Lymphocytes	9.58E-01
Altered T Cell and B Cell Signaling in Rheumatoid Arthritis	9.58E-01
RANK Signaling in Osteoclasts	9.58E-01
Hepatic Fibrosis / Hepatic Stellate Cell Activation	9.42E-01
Fcg Receptor-mediated Phagocytosis in Macrophages and Monocytes	9.18E-01
Chronic Myeloid Leukemia Signaling	9.18E-01
Agranulocyte Adhesion and Diapedesis	9.11E-01
PPAR Signaling	9.11E-01
Role of Macrophages, Fibroblasts and Endothelial Cells in Rheumatoid Arthritis	8.98E-01
Pyrimidine Deoxyribonucleotides De Novo Biosynthesis I	8.78E-01
IL-22 Signaling	8.43E-01
Role of Lipids/Lipid Rafts in the Pathogenesis of Influenza	8.26E-01
Role of JAK family kinases in IL-6-type Cytokine Signaling	8.26E-01
NGF Signaling	8.20E-01
iCOS-iCOSL Signaling in T Helper Cells	8.14E-01
NAD Salvage Pathway II	8.11E-01

Renin-Angiotensin Signaling	8.08E-01
LPS/IL-1 Mediated Inhibition of RXR Function	7.76E-01
Pyrimidine Ribonucleotides Interconversion	7.67E-01
IL-6 Signaling	7.66E-01
p38 MAPK Signaling	7.60E-01
CD28 Signaling in T Helper Cells	7.54E-01
4-1BB Signaling in T Lymphocytes	7.41E-01
Pyrimidine Ribonucleotides De Novo Biosynthesis	7.41E-01
Cytotoxic T Lymphocyte-mediated Apoptosis of Target Cells	7.29E-01
FXR/RXR Activation	7.05E-01
IL-9 Signaling	7.05E-01
Oncostatin M Signaling	7.05E-01
B Cell Development	7.05E-01
TWEAK Signaling	7.05E-01
Inhibition of Angiogenesis by TSP1	7.05E-01
Role of JAK2 in Hormone-like Cytokine Signaling	6.94E-01
Triacylglycerol Biosynthesis	6.94E-01
Complement System	6.72E-01
April Mediated Signaling	6.62E-01
Docosahexaenoic Acid (DHA) Signaling	6.52E-01
Aryl Hydrocarbon Receptor Signaling	6.43E-01
B Cell Activating Factor Signaling	6.42E-01
FcgRIIB Signaling in B Lymphocytes	6.33E-01
Mechanisms of Viral Exit from Host Cells	6.33E-01
Tec Kinase Signaling	5.68E-01
Hepatic Cholestasis	5.53E-01
CNTF Signaling	5.44E-01
Semaphorin Signaling in Neurons	5.37E-01
Lymphotoxin b Receptor Signaling	5.31E-01
Role of IL-17A in Arthritis	5.31E-01
Role of Cytokines in Mediating Communication between Immune Cells	5.17E-01
EGF Signaling	5.17E-01
Nur77 Signaling in T Lymphocytes	5.11E-01
Induction of Apoptosis by HIV1	4.93E-01
GM-CSF Signaling	4.81E-01
IL-8 Signaling	4.79E-01
ERK5 Signaling	4.75E-01
PCP pathway	4.75E-01
IL-17A Signaling in Airway Cells	4.70E-01
Eicosanoid Signaling	4.70E-01
Non-Small Cell Lung Cancer Signaling	4.64E-01
Hypoxia Signaling in the Cardiovascular System	4.64E-01
RAR Activation	4.61E-01
Angiopoietin Signaling	4.59E-01
Role of PI3K/AKT Signaling in the Pathogenesis of Influenza	4.59E-01
CCR5 Signaling in Macrophages	4.44E-01
Growth Hormone Signaling	4.44E-01

Caveolar-mediated Endocytosis Signaling	4.34E-01
PEDF Signaling	4.34E-01
Chemokine Signaling	4.34E-01
IL-17 Signaling	4.29E-01
LPS-stimulated MAPK Signaling	4.24E-01
Role of BRCA1 in DNA Damage Response	4.02E-01
Acute Myeloid Leukemia Signaling	3.98E-01
Regulation of IL-2 Expression in Activated and Anergic T Lymphocytes	3.98E-01
Role of Osteoblasts, Osteoclasts and Chondrocytes in Rheumatoid Arthritis	3.84E-01
cAMP-mediated signaling	3.84E-01
TR/RXR Activation	3.74E-01
TGF-b Signaling	3.66E-01
Virus Entry via Endocytic Pathways	3.59E-01
Apoptosis Signaling	3.59E-01
IL-1 Signaling	3.52E-01
Colorectal Cancer Metastasis Signaling	3.47E-01
T Cell Receptor Signaling	3.31E-01
p53 Signaling	3.28E-01
Antioxidant Action of Vitamin C	3.25E-01
Cholecystokinin/Gastrin-mediated Signaling	3.19E-01
HGF Signaling	3.07E-01
Pancreatic Adenocarcinoma Signaling	3.04E-01
Sphingosine-1-phosphate Signaling	2.96E-01
Gas Signaling	2.96E-01
Role of Tissue Factor in Cancer	2.93E-01
phagosome formation	2.90E-01
Xenobiotic Metabolism Signaling	2.82E-01
Type II Diabetes Mellitus Signaling	2.75E-01
Ga12/13 Signaling	2.75E-01
PTEN Signaling	2.72E-01
HMGB1 Signaling	2.67E-01
Gai Signaling	2.67E-01
Gustation Pathway	2.65E-01
Atherosclerosis Signaling	2.58E-01
GNRH Signaling	2.47E-01
Gaq Signaling	2.12E-01

**Pathways identified in RNA-seq approach**	**-log(p-value)**
Activation of IRF by Cytosolic Pattern Recognition Receptors	7.54E+00
Interferon Signaling	5.57E+00
Role of Pattern Recognition Receptors in Recognition of Bacteria and Viruses	4.49E+00
Pathogenesis of Multiple Sclerosis	3.54E+00
UVA-Induced MAPK Signaling	2.68E+00
Communication between Innate and Adaptive Immune Cells	2.64E+00
Role of RIG1-like Receptors in Antiviral Innate Immunity	2.13E+00
Role of Hypercytokinemia/hyperchemokinemia in the Pathogenesis of Influenza	2.13E+00

Granulocyte Adhesion and Diapedesis	1.84E+00
Retinoic acid Mediated Apoptosis Signaling	1.83E+00
Agranulocyte Adhesion and Diapedesis	1.77E+00
Allograft Rejection Signaling	1.59E+00
Crosstalk between Dendritic Cells and Natural Killer Cells	1.57E+00
OX40 Signaling Pathway	1.57E+00
Death Receptor Signaling	1.54E+00
Bile Acid Biosynthesis, Neutral Pathway	1.43E+00
Renin-Angiotensin Signaling	1.41E+00
Type I Diabetes Mellitus Signaling	1.40E+00
Regulation of Cytokine Production in Macrophages and T Helper Cells by IL-17A and IL-17F	1.30E+00
Regulation of Cytokine Production in Intestinal Epithelial Cells by IL-17A and IL-17F	1.19E+00
IL-22 Signaling	1.17E+00
Role of JAK1, JAK2 and TYK2 in Interferon Signaling	1.17E+00
Dopamine Degradation	1.17E+00
Role of Lipids/Lipid Rafts in the Pathogenesis of Influenza	1.16E+00
IL-17A Signaling in Gastric Cells	1.16E+00
Role of JAK family kinases in IL-6-type Cytokine Signaling	1.16E+00
IL-15 Production	1.13E+00
Cdc42 Signaling	1.08E+00
IL-9 Signaling	1.03E+00
Oncostatin M Signaling	1.03E+00
Dendritic Cell Maturation	1.03E+00
Role of JAK2 in Hormone-like Cytokine Signaling	1.02E+00
Thyroid Hormone Metabolism II (via Conjugation and/or Degradation)	1.02E+00
Hepatic Fibrosis / Hepatic Stellate Cell Activation	1.01E+00
Antigen Presentation Pathway	9.95E-01
Role of PKR in Interferon Induction and Antiviral Response	9.63E-01
Neuroprotective Role of THOP1 in Alzheimer's Disease	9.63E-01
iNOS Signaling	9.24E-01
Graft-versus-Host Disease Signaling	8.88E-01
Autoimmune Thyroid Disease Signaling	8.80E-01
CNTF Signaling	8.56E-01
Role of IL-17A in Arthritis	8.41E-01
Thrombopoietin Signaling	8.34E-01
EGF Signaling	8.26E-01
Melatonin Degradation I	8.19E-01
Superpathway of Melatonin Degradation	7.86E-01
GM-CSF Signaling	7.86E-01
Role of JAK1 and JAK3 in gc Cytokine Signaling	7.79E-01
Protein Ubiquitination Pathway	7.78E-01
Serotonin Degradation	7.73E-01
Hypoxia Signaling in the Cardiovascular System	7.67E-01
Role of PI3K/AKT Signaling in the Pathogenesis of Influenza	7.61E-01
IL-10 Signaling	7.49E-01
CCR5 Signaling in Macrophages	7.43E-01
Growth Hormone Signaling	7.43E-01

Role of MAPK Signaling in the Pathogenesis of Influenza	7.43E-01
IL-3 Signaling	7.32E-01
T Helper Cell Differentiation	7.32E-01
Chemokine Signaling	7.32E-01
Glucocorticoid Receptor Signaling	7.27E-01
JAK/Stat Signaling	7.27E-01
Prolactin Signaling	7.21E-01
TREM1 Signaling	7.16E-01
FLT3 Signaling in Hematopoietic Progenitor Cells	7.16E-01
PDGF Signaling	7.01E-01
VDR/RXR Activation	6.96E-01
Role of BRCA1 in DNA Damage Response	6.96E-01
TGF-b Signaling	6.53E-01
Altered T Cell and B Cell Signaling in Rheumatoid Arthritis	6.49E-01
T Cell Receptor Signaling	6.12E-01
Pancreatic Adenocarcinoma Signaling	5.79E-01
p38 MAPK Signaling	5.42E-01
Gustation Pathway	5.30E-01
FXR/RXR Activation	5.13E-01
D-myo-inositol (1,4,5,6)-Tetrakisphosphate Biosynthesis	5.04E-01
D-myo-inositol (3,4,5,6)-tetrakisphosphate Biosynthesis	5.04E-01
IL-12 Signaling and Production in Macrophages	4.91E-01
D-myo-inositol-5-phosphate Metabolism	4.63E-01
3-phosphoinositide Degradation	4.61E-01
3-phosphoinositide Biosynthesis	4.36E-01
Tec Kinase Signaling	4.36E-01
Hepatic Cholestasis	4.27E-01
Acute Phase Response Signaling	4.13E-01
Production of Nitric Oxide and Reactive Oxygen Species in Macrophages	3.92E-01
IL-8 Signaling	3.84E-01
ERK/MAPK Signaling	3.79E-01
Superpathway of Inositol Phosphate Compounds	3.65E-01
Systemic Lupus Erythematosus Signaling	3.27E-01
Colorectal Cancer Metastasis Signaling	3.05E-01
Role of Macrophages, Fibroblasts and Endothelial Cells in Rheumatoid Arthritis	2.37E-01
