# Supplementary material for: Gene-Expression Profiling Suggests Impaired Signaling via the Interferon Pathway in Cstb-/- Microglia
Source: PLoS One. 2016 Jun 29;11(6):e0158195. doi: 10.1371/journal.pone.0158195 (PMC4927094; doi:10.1371/journal.pone.0158195)
Supplement: S7 Table — (PDF) [file pone.0158195.s010.pdf]

Supplementary table 7: Regulators enriched in differentially expressed genes in *Cstb* $^{-/-}$ microglia.

Upstream regulator identified in microarray approach	Activation z-score	p-value of overlap
TRIM24	5.9	1.20E-56
Ifnar	-5.3	6.50E-53
IRF7	-5.5	4.49E-52
IRF3	-5.4	7.07E-52
STAT1	-5.2	2.51E-36
ACKR2	4.5	1.05E-35
IFNB1	-4.3	2.24E-34
IFNG	-5.7	4.37E-33
IFNL1	-4.6	2.40E-32
IFNA2	-4.7	3.20E-32
IFNAR1	-3.4	2.50E-31
MAVS	-4.2	6.63E-29
IRF5	-4.0	4.10E-28
PRL	-4.8	1.12E-27
DDX58	-3.5	4.98E-27
TICAM1	-4.5	1.89E-26
MAPK1	5.2	5.21E-26
TLR3	-3.9	7.70E-25
SOCS1	3.9	3.70E-23
PTGER4	4.4	1.32E-22
FADD	-3.0	1.87E-21
DNASE2	2.6	2.04E-21
IL1RN	4.0	6.73E-20
TLR4	-3.5	7.69E-20
SASH1	-3.9	1.29E-19
TGM2	-4.6	1.73E-19
IFNLR1		2.88E-19
SAMSN1	-3.9	4.10E-18
TLR9	-4.1	5.31E-18
MYD88	-3.9	7.00E-18
Interferon alpha	-4.1	2.30E-17
NFATC2	-3.7	4.90E-17
IL10RA	4.6	6.25E-17
FZD9	-3.0	1.66E-16
STAT2	-1.6	5.92E-16
IFN alpha/beta	-3.4	1.05E-15
mir-21	3.9	1.77E-15
STAT3	-2.1	5.72E-15
DOCK8	-3.5	7.89E-15
EIF2AK2	-3.4	1.92E-14
TNF	-5.0	2.89E-14
STAT4	-1.4	3.19E-14

Irgm1	3.2	6.42E-14
CNOT7		1.28E-13
Ifna	-2.6	3.75E-13
IKBKE	-1.4	5.85E-13
TCR		1.16E-12
TRIM21	2.4	1.78E-11
IFNA1/IFNA13	-2.4	1.78E-11
IL12 (complex)	-1.1	1.97E-11
IL6	-2.6	2.69E-11
RELA	-2.9	3.77E-11
IKBKG	-2.6	1.01E-10
SOCS3	1.9	2.28E-10
IFIH1	-2.0	2.40E-10
IL12B	-2.8	3.29E-10
TBK1	-1.6	3.88E-10
IRF4	1.3	6.42E-10
IL27	-2.2	6.42E-10
Nr1h	1.1	2.43E-09
NCOA2	-1.3	3.56E-09
IL12A	-2.4	3.56E-09
PLK4	-1.6	6.39E-09
IRF9		6.39E-09
PLK2	-1.6	8.26E-09
IRAK4	-1.4	9.87E-09
PAF1	-2.6	1.16E-08
NFkB (complex)	-3.6	1.17E-08
KDM6B		1.32E-08
JAK1	-2.4	1.34E-08
STAT6	1.3	1.63E-08
BAK1	-2.4	1.68E-08
NLRC5	-1.6	1.98E-08
TREM1	-0.6	2.26E-08
GAPDH	2.4	3.16E-08
TNK1	-2.2	4.03E-08
Map3k7	-1.6	5.26E-08
USP18	2.2	5.56E-08
IFNAR2		7.51E-08
MAPKAP1	-2.0	8.22E-08
CHUK	-3.0	1.04E-07
CD40	-2.4	1.18E-07
NR1H2	0.5	1.45E-07
WHSC1	-2.0	1.47E-07
IRF2	0.3	1.50E-07
TAB1	2.2	2.13E-07
IgG	-0.1	2.31E-07
BAX	-2.4	3.05E-07
RARA	0.3	3.42E-07

IKBKB	-3.1	3.92E-07
SLC9A3		5.02E-07
STAR	2.0	5.71E-07
IFNA4	-2.0	5.71E-07
TLR7	-2.6	6.96E-07
RPSA	2.2	7.31E-07
UBA7		8.19E-07
IFN type 1	-2.0	8.20E-07
LDL	-2.0	1.38E-06
IRF1	-2.2	1.39E-06
MAP3K7	2.0	1.43E-06
IFNGR1		1.94E-06
ISGF3		2.04E-06
CREBBP		2.07E-06
NR3C1	2.6	2.20E-06
NFKB1	-2.2	2.43E-06
MSR1		2.68E-06
PARP1	-2.4	2.68E-06
CD40LG	-2.2	3.16E-06
IL4	0.8	3.31E-06
ISG15		4.06E-06
VIPR1		4.06E-06
SOX11	0.9	4.48E-06
NLRX1		7.07E-06
PLAU	-2.2	7.78E-06
NOS2	-2.1	9.04E-06
RNA polymerase II		9.62E-06
CCL5	-2.2	9.63E-06
S100A8	1.2	9.70E-06
ERK1/2	0.7	9.83E-06
GAB1		1.68E-05
IL17D		1.68E-05
C3AR1	0.9	1.89E-05
Histone h4		2.37E-05
TNFRSF1A	-1.8	2.51E-05
TGFB1	0.7	2.70E-05
LECT2		3.28E-05
Iga		3.28E-05
CCL3L3		3.28E-05
COLEC12		3.51E-05
SCARA5		3.51E-05
SCARA3		3.51E-05
MAP2K3	-2.0	3.78E-05
EBI3	-2.0	3.78E-05
TMEM173	-2.0	3.78E-05
NFKBIA	-1.0	4.28E-05
SATB1	0.1	4.37E-05

NR1H3	0.0	5.33E-05
IRF8	-2.0	5.72E-05
IL1B	-3.1	5.78E-05
ABCA1		6.08E-05
Alpha catenin	2.4	7.21E-05
CSF2	-2.1	7.45E-05
CDKN2A	-2.0	8.30E-05
IL33	-2.0	8.37E-05
CD3	2.6	8.37E-05
S100A9		8.88E-05
CFTR	1.1	9.09E-05

**Upstream regulator identified in RNA-seq approach**	**Activation z-score**	**p-value of overlap**
IRF3	-4.8	2.44E-42
Ifnar	-4.3	4.69E-42
IRF7	-4.5	3.66E-38
ACKR2	4.1	1.46E-35
TRIM24	4.3	1.60E-31
PTGER4	4.0	1.54E-30
IFNA2	-4.1	2.41E-30
IFNB1	-3.5	2.58E-30
IFNL1	-4.0	6.89E-29
MAVS	-3.8	1.23E-28
TLR3	-2.8	4.55E-27
DDX58	-3.1	2.01E-26
IFNAR1	-3.1	2.64E-26
STAT1	-4.1	1.02E-25
SOCS1	3.7	5.88E-24
DNASE2	2.2	6.52E-24
TICAM1	-3.4	2.55E-23
MAPK1	4.2	5.96E-23
FZD9	-2.4	1.25E-22
IRF5	-3.4	1.59E-22
IFNG	-4.1	3.02E-21
Interferon alpha	-3.8	2.62E-20
TLR9	-3.2	4.32E-19
STAT2	-1.5	6.85E-18
Irgm1	3.2	1.17E-17
TLR4	-2.3	5.67E-17
IKBKE	-1.4	2.62E-16
IL1RN	3.3	2.98E-16
IFNLR1		1.94E-15
SASH1	-3.2	2.66E-15
EIF2AK2	-3.1	4.11E-15
TCR		4.13E-15
SAMSN1	-3.2	2.51E-14

PRL	-3.5	1.13E-13
NFATC2	-3.2	1.28E-13
DOCK8	-3.0	1.48E-13
TBK1	-1.5	1.93E-13
Ifna	-2.4	4.08E-13
IKBKG	-2.1	4.30E-13
TNK1	-2.4	2.92E-12
MYD88	-2.3	3.03E-12
NCOA2	-2.4	9.85E-12
PAF1	-2.6	3.28E-11
IFNA4	-2.2	7.88E-11
IFIH1	-1.8	8.69E-11
IFN type 1	-0.2	1.28E-10
TNF	-2.0	1.43E-10
Map3k7	-1.8	1.53E-10
CNOT7		1.69E-10
IFN alpha/beta	-2.6	1.74E-10
IL10RA	2.7	2.38E-10
TGM2	-3.1	2.67E-10
FADD	-2.6	5.96E-10
IFNAR2		1.14E-09
STAT6	1.9	1.30E-09
SOCS3	1.7	1.81E-09
CHUK	-2.1	1.98E-09
IKBKB	-1.6	2.68E-09
STAT3	-1.7	2.73E-09
MAPKAP1	-2.0	2.88E-09
TAB1	2.2	3.28E-09
WHSC1	-2.0	5.18E-09
IL12 (complex)		7.50E-09
STAT4		7.89E-09
IFNA1/IFNA13	-2.0	1.35E-08
NOS2	-1.4	1.51E-08
MAP3K7	2.2	2.27E-08
UBA7		6.70E-08
MSR1		9.64E-08
USP18	1.1	1.24E-07
ISGF3		1.67E-07
IL17A	-0.6	4.19E-07
RARA	0.4	8.74E-07
BAK1	-2.0	9.40E-07
mir-21	2.4	1.12E-06
RELA	-1.0	1.33E-06
GAPDH	2.0	1.41E-06
TNNI3		1.99E-06
TLR7	-2.2	2.66E-06
IFNL3		2.73E-06

KDM6B		5.99E-06
BAX	-2.0	6.25E-06
IL4	1.6	6.64E-06
MKNK2		6.67E-06
MLST8		6.67E-06
LAMA5		9.18E-06
FABP5		9.18E-06
TREM1	1.2	1.10E-05
CCR2	0.6	1.70E-05
HERC5		2.00E-05
MAPK9	0.3	2.61E-05
PARP1	-0.9	2.65E-05
MMP14		2.86E-05
TRPV4		3.27E-05
NFKB1		3.68E-05
IgG	0.7	3.93E-05
MB21D1		3.99E-05
CD5L		3.99E-05
Ifna4		3.99E-05
RETNLB	0.2	4.17E-05
IGHM		4.18E-05
IRF4	0.7	4.40E-05
SLC9A3		4.70E-05
RICTOR	-2.4	5.61E-05
RPSA		5.84E-05
TMEM173		7.89E-05
CD14		9.48E-05
EP300		9.71E-05
PDLIM2	-1.0	9.78E-05
RARRES3		9.93E-05
SARM1		9.93E-05
MTORC2		9.93E-05
ISG15		9.93E-05
TREX1		9.93E-05
